# Tissue-specific and mosaic imprinting defects underlie opposite congenital growth disorders in mice

**DOI:** 10.1371/journal.pgen.1007243

**Published:** 2018-02-22

**Authors:** Andrea Freschi, Stella K. Hur, Federica Maria Valente, Folami Y. Ideraabdullah, Angela Sparago, Maria Teresa Gentile, Andrea Oneglia, Diego Di Nucci, Luca Colucci-D’Amato, Joanne L. Thorvaldsen, Marisa S. Bartolomei, Andrea Riccio, Flavia Cerrato

**Affiliations:** 1 Department of Environmental Technologies, Biological and Pharmaceutical Sciences, University of Campania, “Luigi Vanvitelli”, Naples, Italy; 2 Epigenetics Institute, Department of Cell & Developmental Biology, Perelman School of Medicine at the University of Pennsylvania, Philadelphia, Pennsylvania, United States of America; 3 Department of Genetics, School of Medicine, University of North Carolina, Chapel Hill, North Carolina, United States of America; 4 Department of Nutrition, Gillings School of Public Health, University of North Carolina, Chapel Hill, North Carolina, United States of America; 5 Institute of Genetics and Biophysics, “Adriano Buzzati Traverso” - CNR, Naples, Italy; 6 Department of Experimental Medicine, University of Campania, “Luigi Vanvitelli”, Naples, Italy; University of Bath, UNITED KINGDOM

## Abstract

Differential DNA methylation defects of *H19/IGF2* are associated with congenital growth disorders characterized by opposite clinical pictures. Due to structural differences between human and mouse, the mechanisms by which mutations of the *H19/IGF2* Imprinting Control region (IC1) result in these diseases are undefined. To address this issue, we previously generated a mouse line carrying a humanized IC1 (hIC1) and now replaced the wildtype with a mutant IC1 identified in the overgrowth-associated Beckwith-Wiedemann syndrome. The new humanized mouse line shows pre/post-natal overgrowth on maternal transmission and pre/post-natal undergrowth on paternal transmission of the mutation. The mutant hIC1 acquires abnormal methylation during development causing opposite *H19/Igf2* imprinting defects on maternal and paternal chromosomes. Differential and possibly mosaic *Igf2* expression and imprinting is associated with asymmetric growth of bilateral organs. Furthermore, tissue-specific imprinting defects result in deficient liver- and placenta-derived *Igf2* on paternal transmission and excessive *Igf2* in peripheral tissues on maternal transmission, providing a possible molecular explanation for imprinting-associated and phenotypically contrasting growth disorders.

## Author summary

A humanized mouse line carrying a mutation of the *H19/IGF2* imprinting control region demonstrates how tissue-specific and mosaic imprinting alterations result in growth disorders with opposite clinical pictures and asymmetric growth of bilateral organs.

## Introduction

Imprinted genes show monoallelic and parent-of-origin-dependent expression and play key roles in the control of growth and development. In humans, altered expression of imprinted genes is associated with Imprinting Disorders (IDs) that are characterized by growth, metabolic and behavioural disturbances [1–2]. Most imprinted genes are organized in clusters, in which their parental-specific expression is dependent on Imprinting Control Regions (ICRs). ICRs correspond to 2–5 kb-long sequences with differential DNA methylation on their maternal and paternal alleles. Parental-specific ICR methylation is acquired during gametogenesis and maintained in the zygote and somatic cells throughout development despite extensive demethylation occurring in the embryo before implantation and *de novo* methylation after implantation [3]. An evolutionary conserved cluster of imprinted genes of about 1 Mbp is located on human chromosome 11p15.5 and mouse distal chromosome 7. The cluster is organized in two functionally independent domains, each with its own ICR. In the telomeric domain, the *H19/IGF2* intergenic differentially methylated region (also known as and herein termed Imprinting Center 1, IC1) controls the reciprocal imprinting of the maternally expressed *H19* and paternally expressed Insulin like Growth Factor 2 (*IGF2*) genes [3]. IGF2 is required for normal foetal growth [4]. The liver is the main endocrine source of IGF2 in post-natal life, but autocrine/paracrine activity is found in most embryonic tissues, particularly in placenta, where it is needed for correct allocation of maternal resources to fetal growth [5, 6]. *H19* is a long non-coding RNA with inhibitory activity on foetal growth [7]. Both *IGF2/Igf2* and *H19* are down-regulated after birth in both humans and mice but their deficiencies have long-lasting effects on somatic growth [4, 7–11].

**Fig 1:**
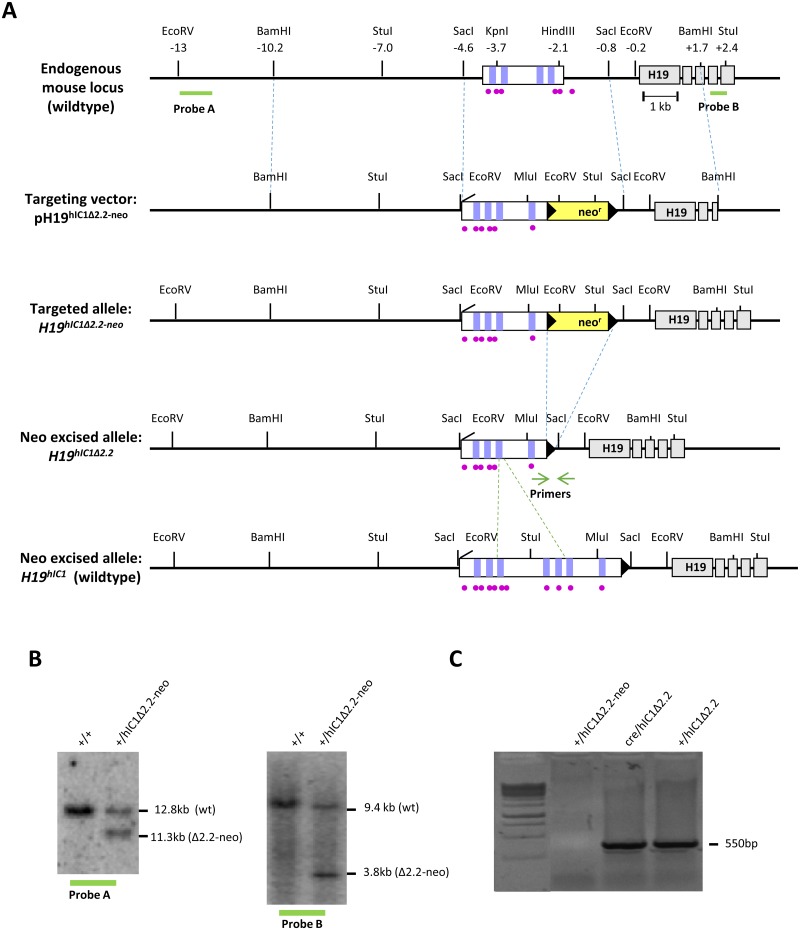
Strategy for generating the *H19*^*hIC1*Δ*2*.*2*^ allele. (A)Schematics of the endogenous mouse *H19* locus, targeting vector for mutant hIC1 (pH19^hIC1Δ2.2-neo^), targeted mutant allele with the neomycin resistance cassette (*H19*^*hIC1*Δ*2*.*2-neo*^), targeted mutant allele after neoR excision (*H19*^*hIC1*Δ*2*.*2*^) and targeted wildtype hIC1 allele (*H19*^*hIC1*^). Depicted are the IC1 (white rectangle) with CTCF binding sites (vertical lilac bars within IC1), ZFP57 binding sites (purple circles), *H19* exons (gray rectangles); pBluescriptIIKS vector with the human insert flanked by the mouse sequences (outlined with dashed blue lines) used as arms for the homologous recombination, neoR cassette (yellow rectangle), loxP sites (black arrowheads). Restriction sites and their relative positions (in kb) to the 5’ end of *H19* are also indicated. Probes (A and B) used for Southern blot analysis are indicated as thick green lines below the endogenous locus. Positions of the primers for testing the neo cassette excision are indicated by green arrows. Dashed green lines define the region of hIC1 deleted in the hIC1Δ2.2 allele. (B) Southern blot analysis to confirm targeting of mutant hIC1 allele. DNA from wildtype (+/+) and targeted (+/hIC1Δ2.2-neo) ES cells was digested with *Eco*RV and hybridized to probe A (lying 5’ of the targeting arm) or digested with *Stu*I and hybridized to probe B (lying 3’ of the targeting arm). (C) Confirmation of Cre-mediated excision of neoR cassette. Excision was confirmed by PCR with primers (green arrows in fig 1A) flanking neoR. A PCR product of 550 bp confirming excision was obtained from genomic DNA of F1 (Cre/hIC1Δ2.2) mice, and F2 (+/hIC1Δ2.2) derived from F1 crossed to wildtype mice. No PCR product was obtained from KI mice (+/hIC1Δ2.2-neo) with the NeoR cassette.

In mouse embryos, *H19* and *Igf2* are co-expressed in endoderm- and mesoderm-derived tissues, and their expression depends on the same downstream enhancers on the maternal and paternal chromosomes, respectively [12–15]. IC1 is structurally different in humans and mice—human IC1 (hIC1) is ~ 5kb-long and contains seven CCCTC-binding factor (CTCF) target sites (CTS), whereas mouse IC1 (mIC1) is ~ 2kb-long and contains four CTS (Fig 1A). CTCF binding to IC1 is required for the formation of an insulator with enhancer blocking activity in both species [16–17]. Because IC1 is methylated on the paternal allele and CTCF binding is inhibited by DNA methylation, the insulator is formed only on the maternal chromosome where it prevents the activation of *IGF2* but allows activation of *H19* by the enhancers. The opposite happens on the paternal chromosome, where *IGF2* is activated and *H19* silenced.

Opposite hIC1 methylation and imprinting defects are associated with the Beckwith-Wiedemann syndrome (BWS, MIM #130650) and the Silver Russell syndrome (SRS, MIM #180860), two IDs characterised by congenital overgrowth and congenital undergrowth, respectively [1]. In particular, hIC1 gain of methylation (GOM) resulting in *IGF2* activation and *H19* repression on the maternal chromosome is found in 5–10% of BWS cases. Conversely, hIC1 loss of methylation (LOM) leading to *IGF2* repression and *H19* activation on the paternal chromosome occurs in about 50% of SRS patients. In a number of BWS cases with hIC1 GOM, small deletions and single nucleotide variations within hIC1 co-segregate with the clinical phenotype and abnormal methylation upon maternal transmission but lead to normal phenotype on paternal transmission [11, 18–25]. Different paternally inherited hIC1 deletions have been recently described in a few cases of SRS with IC1 LOM [26]. Although the extent of these deletions is similar to those found in BWS families, the sequence and the CTSs involved are different.

In the last twenty years, several mutations have been introduced into the endogenous mIC1 locus [27–35]. This work has been instrumental to demonstrate the fundamental role that *H19/IGF2* imprinting has in the aetiology of congenital overgrowth and undergrowth associated with imprinting defects. However, due to its structural differences with the orthologous mouse locus, the mechanism by which hIC1 mutations affect epigenotype and phenotype in both BWS and SRS is still obscure.

To clarify the role of hIC1 mutations in the origin of imprinting defects and in the pathogenesis of BWS and SRS, we generated a knock-in (KI) mouse line in which the endogenous mIC1 was replaced by the orthologous hIC1 allele carrying a mutation (hIC1Δ2.2) that is associated with BWS on maternal transmission [36]. We compared the *H19*^*hIC1*Δ*2*.*2*^ line with wildtype mice and the previously described line carrying a humanized *H19* allele with the wildtype human ICR (*H19*^*hIC1*^) [37]. The results demonstrate growth and molecular abnormalities of the mice with maternal and paternal transmission of the mutant KI that resemble those of BWS and SRS, respectively, including asymmetric organ growth. Importantly, tissue-specific and mosaic dysregulation of *H19*/*Igf2* imprinting indicates new pathogenetic mechanisms of congenital growth disorders and lateralized/regional over/under-growth associated with imprinting defects.

## Results

### Generation of the *H19*^*hIC1*Δ*2*.*2*^ and *H19*^*hIC1*^ lines

In order to study the relationship between genotype, epigenotype and phenotype of IDs in the mouse, we replaced the endogenous mIC1 with a mutant hIC1 allele (*H19*^*hIC1*Δ*2*.*2*^) previously found in BWS [19, 36], by homologous recombination in mouse embryonic stem (ES) cells (Fig 1A). Chimeras were obtained and germ line transmission was confirmed by Southern blotting (Fig 1B). The transgenic line was then bred to pCX-NLS- Cre transgenic line to remove the NeoR cassette and its excision was confirmed by PCR (Fig 1C. See also Materials and methods).

We crossed KI mice on a C56BL/6 (B6) background to Balb/C and used polymorphisms present between these two strains to distinguish parental alleles. To compare the behaviour of the mutant with that of the wildtype hIC1 allele (*H19*^*hIC1*^) in the same genetic background, the previously described *H19*^*hIC1*^ line [37] was generated anew by targeting ES cells and breeding the mice using similar procedures to what done with the *H19*^*hIC1*Δ*2*.*2*^ line. Subsequent experiments were performed on both humanized KI lines. The wildtype +/+ littermates were also assayed as control.

### Maternal transmission of the *H19*^*hIC1*Δ*2*.*2*^ allele results in overgrowth and tissue-specific *Igf2* activation

#### Phenotypic analysis

**Fig 2:**
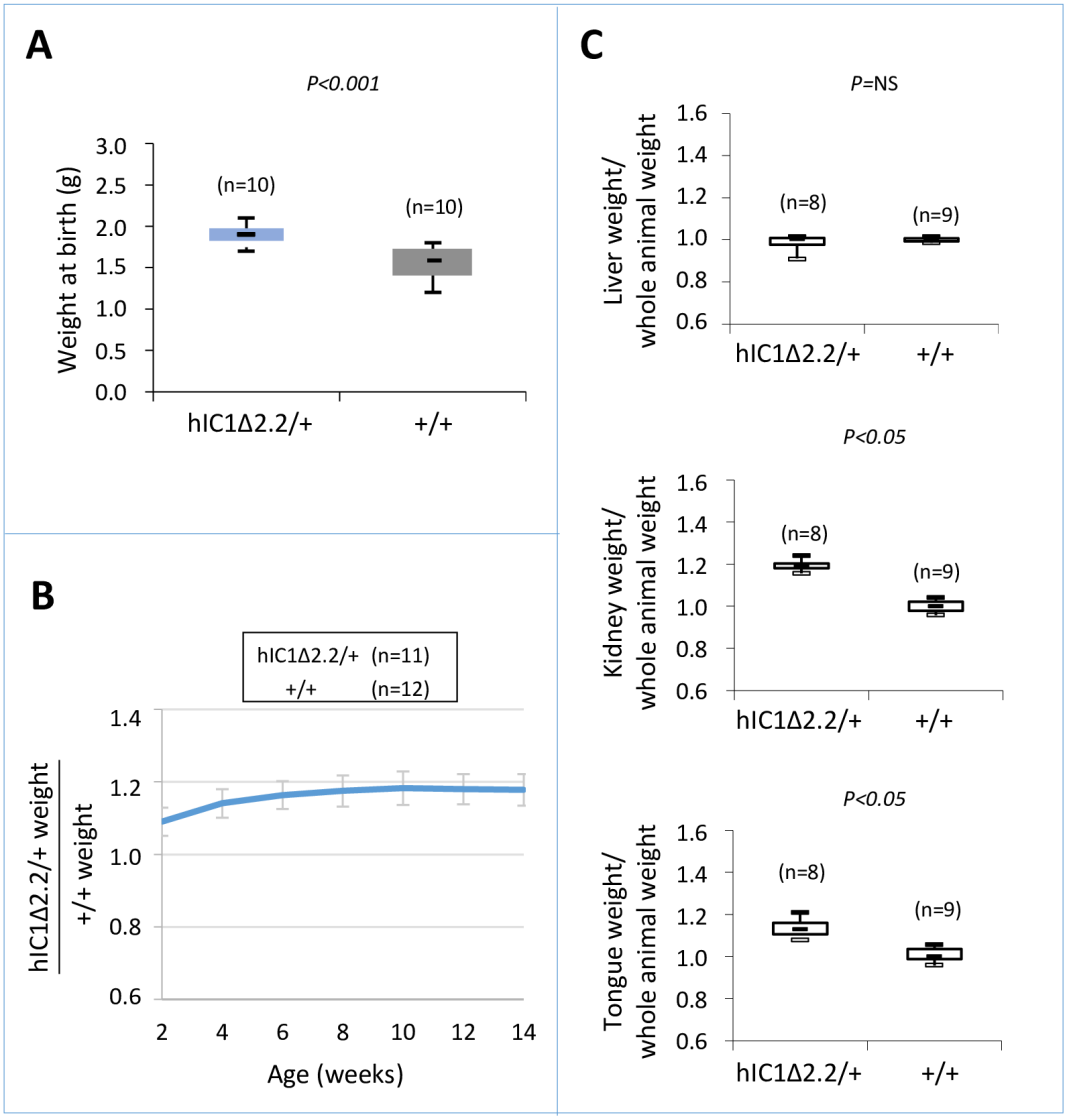
Somatic overgrowth on maternal transmission of the *H19*^*hIC1*Δ*2*.*2*^ allele. Box plot of birth weights (A), growth charts (B), and box plots of organ/whole animal weights at 14 weeks of age (C) of *H19*^*hIC1*Δ*2*.*2/+*^ and *H19*^*+/+*^ littermates. Box plots display the full range of variation (from minimum to maximum values), the likely range of variation (first and third quartiles) and the median (horizontal bar within box). Values in brackets indicate the number of animals derived from three litters that was used for this study. P = P-value calculated by two-tailed Student’s T-test. In (B) the growth chart is expressed as ratio between the weights of KI and weights of +/+ animals in three litters. Bars represent the mean ± SEM. In (C) the box plots represent the relative values of organ weight/whole animal weight ratios, obtained by arbitrarily setting the median value of *H19*^*+/+*^ as 1.

To determine the consequence of maternal transmission of the *H19*^*hIC1*Δ*2*.*2*^ allele on somatic growth, we measured the body weight of the F1 progeny of *H19*^*hIC1*Δ*2*.*2/+*^ X *H19*^*+/+*^ crosses from birth to 14 weeks of age. Compared to their *H19*^*+/+*^ littermates, *H19*^*hIC1*Δ*2*.*2/+*^ mice were significantly heavier, longer and larger at birth and overgrowth persisted postnatally until adult age (Fig 2A and 2B). In addition, kidney and tongue but not liver were significantly heavier in adult *H19*^*hIC1*Δ*2*.*2/+*^ mice, if normalized to the weight of the whole animal, indicating the presence of nephromegaly and macroglossia (Fig 2C). Consistent with previously published data [37], no growth difference was observed between *H19*^*hIC1/+*^ and *H19*^*+/+*^ mice (S1 Fig). Thus, in contrast to *H19*^*hIC1*^, maternal transmission of *H19*^*hIC1*Δ*2*.*2*^ results in congenital macrosomia and organomegaly that persist in the adulthood.

#### Molecular analysis

**Fig 3:**
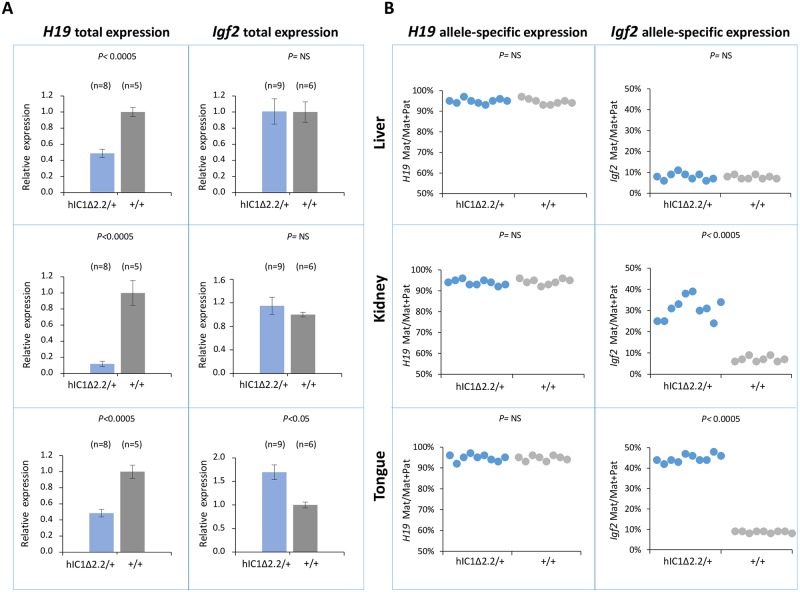
Analysis of *H19* and *Igf2* expression in *H19*^*hIC1*Δ*2*.*2/+*^ newborn mice. (A) Histograms of total *H19* and *Igf2* expression in three different neonatal organs of *H19*^*hIC1*Δ*2*.*2/+*^ and *H19*^*+/+*^ littermates analysed by RT-qPCR. The mean value of *H19*^*+/+*^ is set arbitrarily as 1. NS, Not Significant. Bars represent the mean ± SEM. (B) Allele-specific expression of *H19* and *Igf2*. Dots indicate the percent expression of the maternal allele in each individual sample. The animals used for this study derived from three litters.

To examine expression and methylation of the *H19/Igf2* locus upon maternal transmission of the KI alleles, RNA and DNA were extracted from liver, kidney and tongue of newborn (P1) mice. When measured by quantitative real-time PCR (RT-qPCR), *H19* RNA levels were significantly lower in *H19*^*hIC1*Δ*2*.*2/+*^ mice compared with their *H19*^*+/+*^ littermates in all analyzed tissues, while levels of *Igf2* RNA were higher in tongue, but similar or not significantly increased in liver and kidney (Fig 3A). Allele-specific RNA analysis revealed that, while the imprinted *H19* expression (paternal allele repressed) was normally maintained in all tissues of *H19*^*hIC1*Δ*2*.*2/+*^ mice as well as *H19*^*+/+*^ littermates, the maternal *Igf2* allele was derepressed in tongue (44% mat/mat+pat expression) and kidney (31%) of *H19*^*hIC1*Δ*2*.*2/+*^ mice (Fig 3B). In contrast, the relative expression (8%) of maternal *Igf2* in liver of *H19*^*hIC1*Δ*2*.*2/+*^ and *H19*^*+/+*^ mice was comparable. Consistent with previous data [37], total expression levels and imprinting of both *H19* and *Igf2* in *H19*^*hIC1/+*^ and *H19*^*+/+*^ mice were comparable (S2A and S2B Fig).

**Fig 4:**
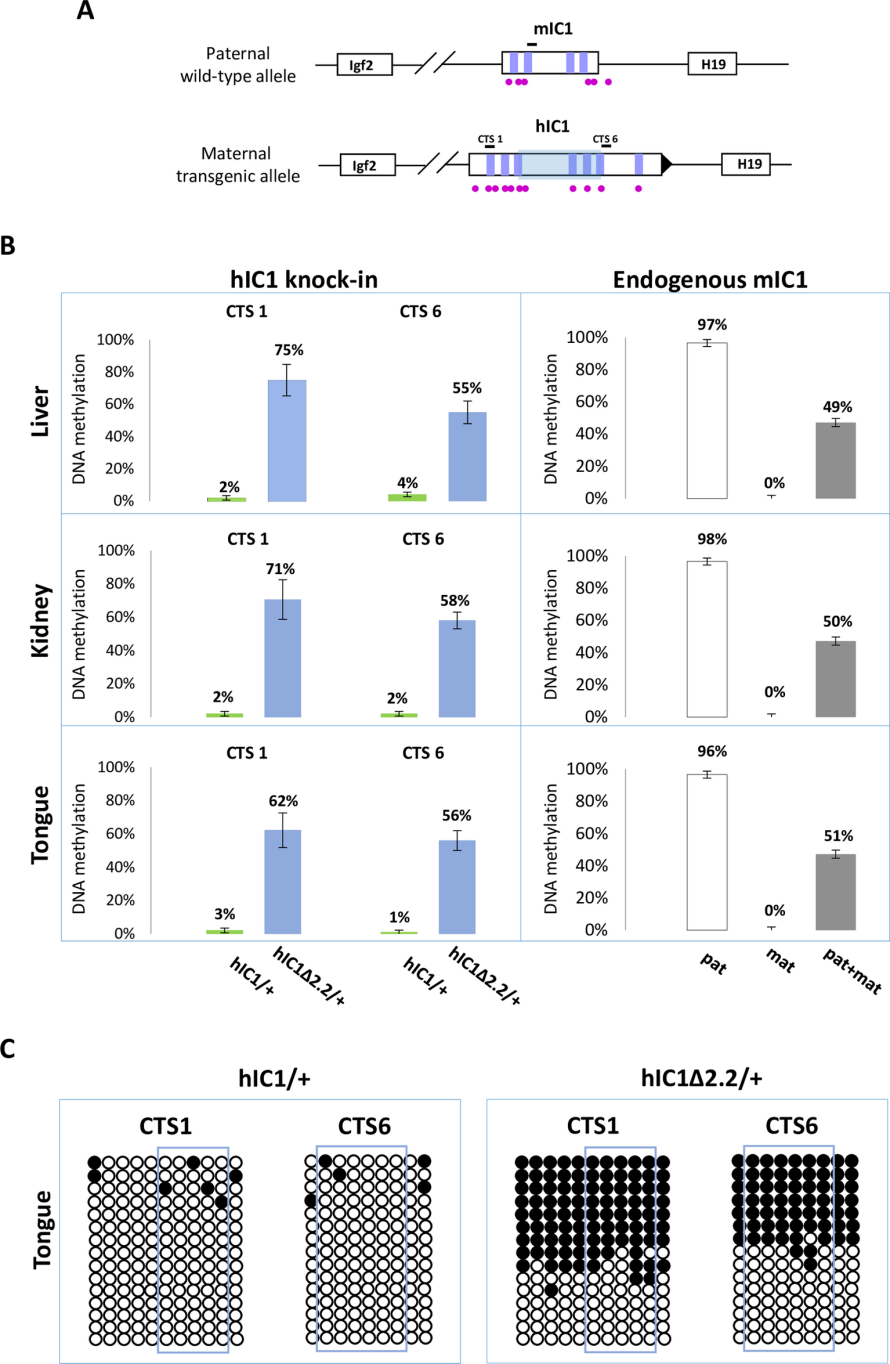
Gain of IC1 methylation in *H19*^*hIC1*Δ*2*.*2/+*^ mice. (A) Graph representing the humanized and endogenous *H19/Igf2* locus with the regions (indicated by black bars) whose methylation was analysed by pyrosequencing. The region deleted in *H19*^*hIC1*Δ*2*.*2*^ is represented by a shaded light blue box. The coordinates of the sequenced CpGs are: CTS1 in hg39—chr11:2,024,193–2,024,261; CTS6 in hg39—chr11:2,021,096–2,021,153; mIC1 in mm9—chr7:149,767,625–149,767,705; mIC2 in mm9—chr7:150,482,365–150,482,486. Other details are described in the legend to Fig 1. (B) Percent methylation measured by pyrosequencing at two CTCF target sites (CTS1 and CTS6) in three different organs collected from *H19*^*hIC1*Δ*2*.*2/+*^ and *H19*^*hIC1/+*^ mice at birth (left panel). Endogenous mIC1 analysed as control of methylation: the paternal allele (pat) was assayed in *H19*^*hIC1*Δ*2*.*2/+*^, the maternal allele (mat) in *H19*^*+/hIC1*Δ*2*.*2*^, and both alleles (pat + mat) in *H19*^*+/+*^ mice (right panel). Each histogram represents the methylation mean value of 5 (CTS1 and mIC1) or 6 (CTS6) CpGs, tested in *H19*^*hIC1*Δ*2*.*2/+*^ (n = 10), *H19*^*hIC1/+*^ (n = 9) and *H19*^*+/+*^ (n = 7) mice derived from three litters. Bars represent the mean ± SEM. Note that methylation (ranging from 55% to 75%) was observed at the *H19*^*hIC1*Δ*2*.*2*^ and not at the *H19*^*hIC1*^ allele. (C) IC1 methylation analysed by bisulphite treatment followed by cloning and sequencing in tongue of *H19*^*hIC1/+*^ and *H19*^*hIC1*Δ*2*.*2/+*^ mice. Each line corresponds to a single template DNA molecule cloned; each circle corresponds to a CpG dinucleotide. Filled circles designate methylated cytosines; open circles, unmethylated cytosines. CpGs measured by pyrosequencing are framed.

In BWS, maternal transmission of IC1 mutations is associated with IC1 GOM [36]. To investigate if this phenotype was reproduced in the mutant KI mice, we assayed the methylation of two different IC1 regions, CTS1 and CTS6, in *H19*^*hIC1*Δ*2*.*2/+*^ and *H19*^*hIC1/+*^ mice, by pyrosequencing of bisulfite-converted DNA. The results demonstrated that in contrast with *H19*^*hIC1*^ that maintained very low (1–4%) methylation levels (Fig 4A and Ref. 37), *H19*^*hIC1*Δ*2*.*2*^ was aberrantly methylated at similar levels (62–75% in CTS1 and 55–58% in CTS6) in liver, kidney and tongue. As expected, the endogenous paternal mIC1 was fully methylated and the maternal mIC1 was unmethylated. Hypermethylation of *H19*^*hIC1*Δ*2*.*2*^ was confirmed by bisulfite sequencing. (Fig 4B). This assay shows complete methylation of 7 out of 15 molecules indicating epigenetic mosaicism. Methylation analysis was repeated in three consecutive generations of *H19*^*hIC1*Δ*2*.*2/+*^ mice with consistent results (S3 Fig).

In summary, *H19*^*hIC1*Δ*2*.*2*^ exhibits aberrant hIC1 methylation upon maternal transmission and is associated with abnormal *H19* repression and tissue-specific *Igf2* activation.

### Paternal transmission of the *H19*^*hIC1*Δ*2*.*2*^ allele results in growth restriction and tissue-specific *Igf2* repression

#### Phenotypic analysis

**Fig 5:**
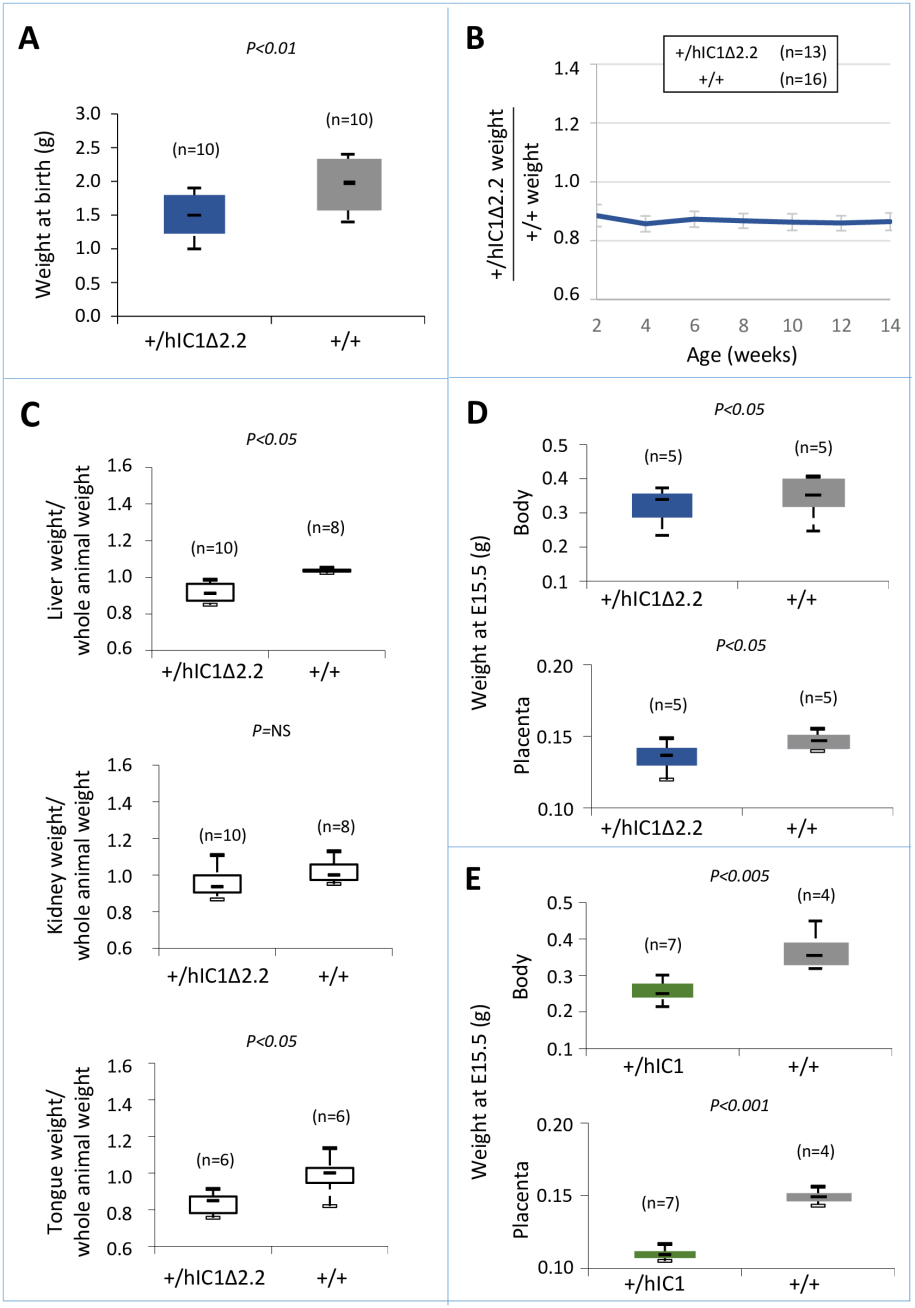
Somatic undergrowth on paternal transmission of the *H19*^*hIC1*Δ*2*.*2*^ allele. (A-C) Box plot of birth weights (A), growth charts (B), and box plots of organ weights at 14 weeks of age (C) of *H19*^*+/hIC1*Δ*2*.*2*^ and *H19*^*+/+*^ littermates. Box plots in (A) and (C) and growth chart in (B) are as in Fig 2. (D-E) Box plot of embryo body and placenta weights of *H19*^*+/hIC1*Δ*2*.*2*^ (D) and *H19*^*+/hIC1*^ (E) mice at E15.5 compared with *H19*^*+/+*^ littermates. The animals used for this study derived from three (A-C) or two litters (D-E).

We next assessed the effect of paternal transmission of the *H19*^*hIC1*Δ*2*.*2*^ allele. Paternal transmission of *H19*^*hIC1*^ resulted in embryonic lethality, confirming our previous data [37]. In contrast, the F1 progeny of heterozygous *H19*^*hIC1*Δ*2*.*2*^ males bred to *H19*^*+/+*^ females presented *H19*^*+/+*^ and KI pups in 1:1 ratio. However, compared with their *H19*^*+/+*^ littermates, *H19*^*+/hIC1*Δ*2*.*2*^ mice weighed less and were smaller at birth and maintained growth restriction postnatally until adult age (Fig 5A and 5B). *H19*^*+/hIC1*Δ*2*.*2*^ mice also appeared very lean and the weight of their liver and tongue, normalized to that of the whole animal, was significantly lower than that of *H19*^*+/+*^ mice (Fig 5C).

To compare the effects of paternal transmission of *H19*^*hIC1*^ and *H19*^*hIC1*Δ*2*.*2*^ on somatic growth, we collected the embryos at a stage (E15.5) preceding the death of *H19*^*+/hIC1*^ mice in utero. Body and placenta of embryos from both KI strains were smaller than *H19*^*+/+*^ littermates. However, *H19*^*+/hIC1*^ embryos were even smaller (30% lighter than *H19*^*+/+*^ littermates) than *H19*^*+/hIC1*Δ*2*.*2*^ embryos (13% lighter than *H19*^*+/+*^ littermates) (Fig 5D). A more dramatic difference was found in the placental weights (Fig 5E, *H19*^*+/hIC1*^ line ~30% lighter than *H19*^*+/+*^ littermates; *H19*^*+/hIC1*Δ*2*.*2*^ line ~8% lighter than *H19*^*+/+*^ littermates).

Thus, although paternal transmission of both *H19*^*hIC1*^ and *H19*^*hIC1*Δ*2*.*2*^ results in growth restriction, the phenotype of *H19*^*+/hIC1*Δ*2*.*2*^ is less severe than that of *H19*^*+/hIC1*^ embryos and is compatible with life.

#### Molecular analysis

**Fig 6:**
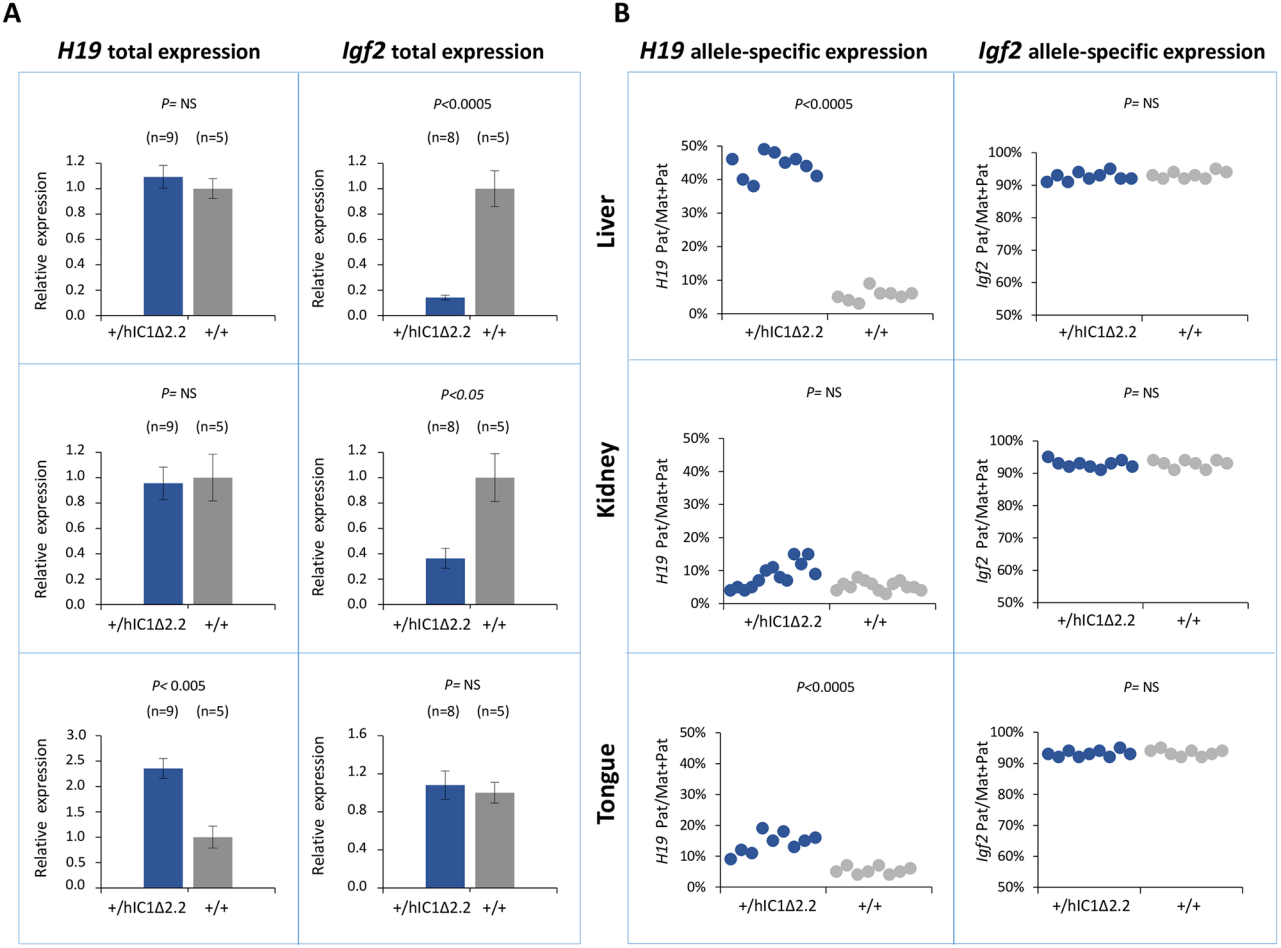
Analysis of *H19* and *Igf2* expression in *H19*^*+/hIC1*Δ*2*.*2*^ newborn mice. (A) Histograms of total *H19* and *Igf2* expression in three different neonatal organs of *H19*^*+/hIC1*Δ*2*.*2*^ and *H19*^*+/+*^ littermates analysed by RT-qPCR. The mean value of *H19*^*+/+*^ is set arbitrarily as 1. NS, Not Significant. Bars represent the mean ± SEM. (B) Dots indicate the percent expression of the paternal allele in each individual sample. The animals used for this study derived from three litters.

Gene expression and DNA methylation at the *H19/Igf2* locus were measured in liver, kidney and tongue of P1 *H19*^*+/hIC1*Δ*2*.*2*^ and *H19*^*+/+*^ littermates. Total *H19* expression was significantly increased in tongue, while *Igf2* levels were severely decreased in liver and more moderately in kidney of *H19*^*+/hIC1*Δ*2*.*2*^ mice (Fig 6A). Analysis of allele-specific expression revealed that in *H19*^*+/hIC1*Δ*2*.*2*^ mice the paternal *H19* allele was strongly derepressed in liver (44% pat/mat+pat expression) and more moderately in tongue (15%, see Fig 6B). In kidney, the relative expression of the paternal *H19* allele (8%) was comparable to that of *H19*^*+/+*^ mice. The imprinted *Igf2* expression (maternal allele repressed) was properly maintained in all three tissues in *H19*^*+/hIC1*Δ*2*.*2*^ as well as their *H19*^*+/+*^ littermates (Fig 6B).

To compare *H19/Igf2* expression of *H19*^*+/hIC1*Δ*2*.*2*^ and *H19*^*+/hIC1*^, E15.5 conceptuses were also analysed. Total *H19* expression was increased about 2-fold in embryo body and placenta of *H19*^*+/hIC1*^, but *H19* activation was less evident and did not reach statistical significance in *H19*^*+/hIC1*Δ*2*.*2*^. In contrast, *Igf2* expression was downregulated in embryo and placenta of both KI lines, although repression in embryos was less dramatic in *H19*^*+/hIC1*Δ*2*.*2*^ than in *H19*^*+/hIC1*^ mice (S4A and S4B Fig). Allele-specific RNA analysis demonstrated that the paternal *H19* allele was strongly activated (close to 50% of total expression) in both embryo body and placenta of E15.5 *H19*^*+/hIC1*^, but only to a lower extent (25% in embryo body and 35% in placenta) in *H19*^*+/hIC1*Δ*2*.*2*^. The imprinted *Igf2* expression was properly maintained in both *H19*^*+/hIC1*^ and *H19*^*+/hIC1*Δ*2*.*2*^ (S4C and S4D Fig and Ref. 37).

**Fig 7:**
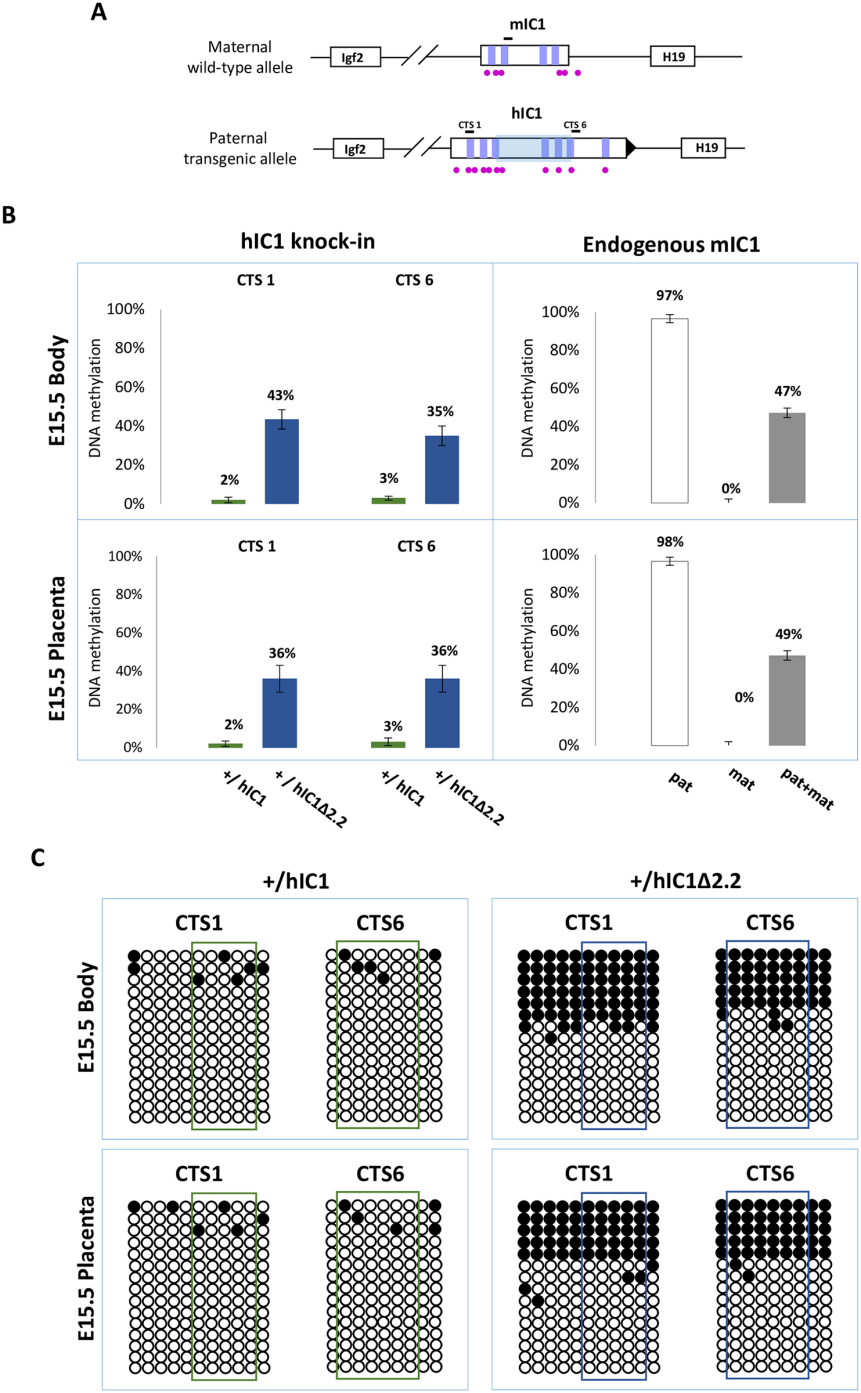
IC1 methylation in *H19*^*+/hIC1*Δ*2*.*2*^ and *H19*^*+/hIC1*^ mice at E15.5. (A) See legend to Fig 4A. (B) Percent methylation measured by pyrosequencing at two CTCF target sites (CTS1 and CTS6) from embryo body and placenta of *H19*^*+/hIC1*Δ*2*.*2*^ and *H19*^*+/hIC1*^ mice at E15.5 (left panel). The endogenous mIC1 was analysed as in Fig 4. Each histogram represents the methylation mean value of 5 (CTS1 and mIC1) or 6 (CTS6) CpGs, tested in two litters of each KI: +/hIC1Δ2.2 (n = 6), *+/+* (n = 5); +/hIC1 (n = 6) and *+/+* (n = 4) embryos. Bars represent the mean ± SEM. (C) IC1 methylation analysed by bisulphite treatment followed by cloning and sequencing in embryo body and placenta of *H19*^*+/hIC1*Δ*2*.*2*^ and *H19*^*+/hIC1*^ mice at E15.5. See legend to Fig 4 for more details.

We then measured IC1 methylation in *H19*^*+/hIC1*Δ*2*.*2*^ and *H19*^*+/hIC1*^ mice at E15.5 by pyrosequencing. In contrast with the extremely low (2–3%) methylation of *H19*^*hIC1*^, the *H19*^*hIC1*Δ*2*.*2*^ allele was found partially (35–43%) methylated in both embryo body and placenta (Fig 7A). Bisulfite-sequencing confirmed the differential methylation of the *H19*^*hIC1*Δ*2*.*2*^ and *H19*^*hIC1*^ alleles (Fig 7B). The analysis of liver, kidney and tongue of P1 mice demonstrated that methylation of the *H19*^*hIC1*Δ*2*.*2*^ allele was maintained postnatally. Methylation levels (64–77%) were similar in these three adult tissues. They were higher than in embryonic tissues, but still lower than the endogenous paternal mIC1 allele that was fully methylated (S5A and S5B Fig). Methylation analysis was repeated in three consecutive generations of KI mice derived from breeding of *H19*^*+/hIC1*Δ*2*.*2*^ males with +/+ females with consistent results (S6A Fig). Also, methylation of the hIC1Δ2.2 allele did not change after the passage from the female to male germline, as demonstrated by the methylation values of mice of two consecutive generations derived from reciprocal breeding (S6B Fig).

In summary, paternal transmission of *H19*^*hIC1*Δ*2*.*2*^ results in IC1 hypomethylation that is less severe than that of *H19*^*hIC1*^ and is associated with an imprinting defect on the paternal chromosome causing moderate *H19* activation accompanied by *Igf2* repression that is particularly prominent in liver and placenta.

### Differential *Igf2* expression and imprinting is associated with kidney asymmetry in mutant KI mice

**Fig 8:**
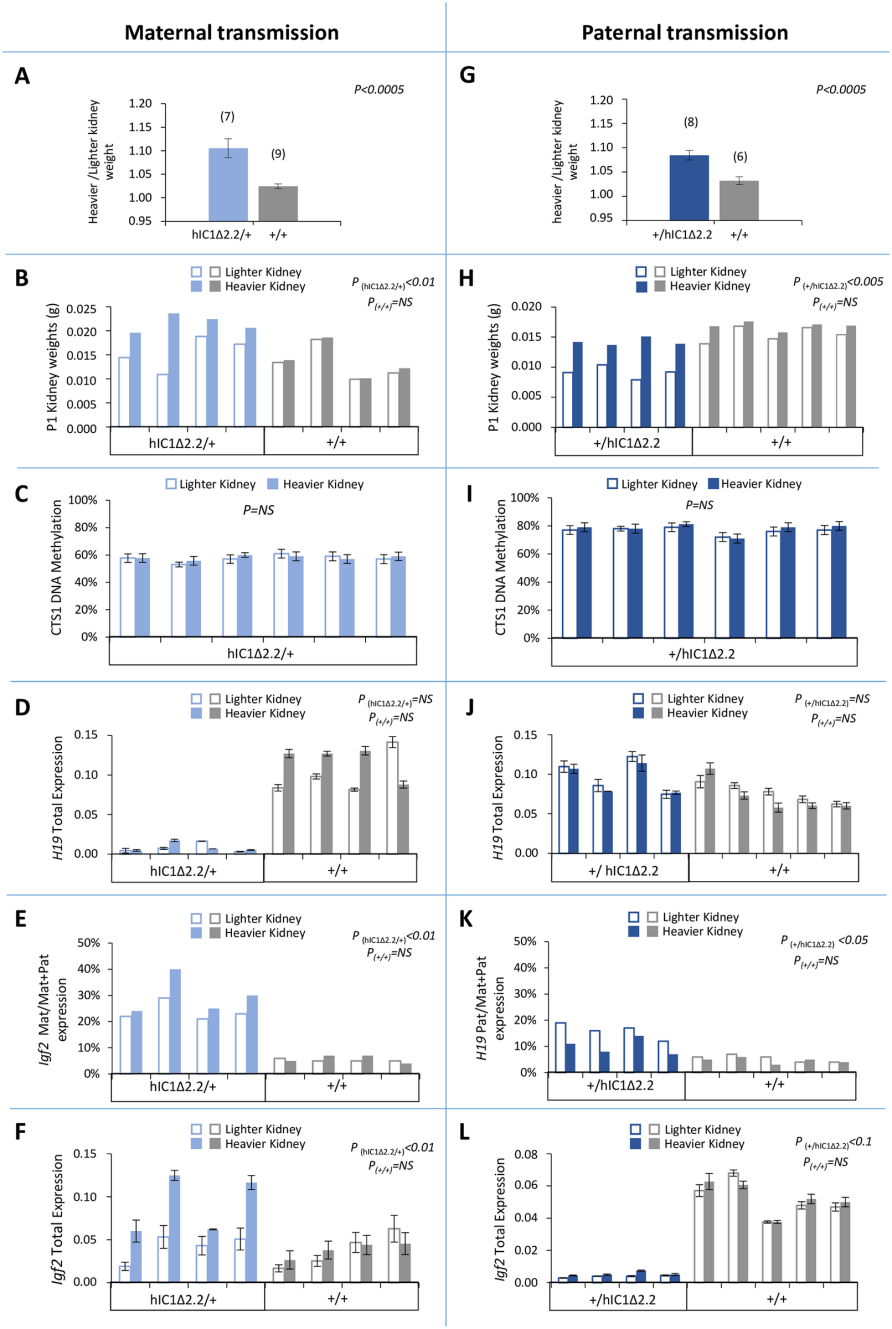
Kidney asymmetry and *H19/Igf2* expression and imprinting on maternal and paternal transmission of the *H19*^*+/hIC1*Δ*2*.*2*^ allele. (A-F) Analysis of maternal transmission. Weight difference of the kidneys at 12 weeks of age (A), kidney weights at birth (B), DNA methylation level of CTS1 (C), total *H19* expression (D), allele-specific *Igf2* expression (E), and total *Igf2* expression (F) in heavier and lighter kidneys of *H19*^*hIC1*Δ*2*.*2/+*^ and *H19*^*+/+*^ mice. (G-L) Analysis of paternal transmission. Weight difference of the kidneys at 12 weeks of age (G), kidney weights at birth (H), DNA methylation level of CTS1 (I), total *H19* expression (J), allele-specific *H19* expression (K), and total *Igf2* expression (L) analysed in heavier and lighter kidneys of *H19*^*+/hIC1*Δ*2*.*2*^ and *H19*^*+/+*^ mice. Bars in A and G represent the mean ± SEM. Bars in C-F and I-L represent the mean ± SEM of three technical replicates of the same sample. P-value calculated by two-tailed Student’s T-test on lighter vs heavier kidneys of littermates with the same genotype. The animals used for this study derived from two litters. Note that differences in weight, *Igf2* expression and *H19/Igf2* imprinting were present only in mice carrying the *H19*^*hIC1*Δ*2*.*2*^ allele.

To investigate the presence of kidney asymmetry, a clinical sign often found in BWS [2], we measured the weight of left and right kidneys of the mice carrying the mutant KI. Maternal transmission was first assessed. A significant difference between the two kidneys (with no bias toward the left or right organ) was found in adult and newborn *H19*^*hIC1*Δ*2*.*2/+*^ mice, but not in *H19*^*+/+*^ littermates (Fig 8A and 8B). In contrast, no difference was observed in *H19*^*hIC1/+*^ mice (S7 Fig). hIC1 methylation and *H19/Igf2* expression were then assessed in the organs of the neonates carrying the mutant KI. While DNA methylation and *H19* expression were comparable (Fig 8C and 8D), significant differences of total and allele-specific expression of *Igf2* were found between the heavier and lighter kidneys of *H19*^*hIC1*Δ*2*.*2/+*^ (with higher expression in the larger organ) mice (Fig 8E and 8F). Next, we investigated the presence of kidney asymmetry in the mice with paternal transmission of the KI. As for maternal transmission, weight differences between the two kidneys in *H19*^*+/hIC1*Δ*2*.*2*^ mice were significantly higher than in their *H19*^*+/+*^ littermates, both at neonatal and adult stages (Fig 8G and 8H). Also, comparable hIC1 methylation and global *H19* RNA levels were found in left and right kidneys of all tested animals (Fig 8I and 8J). In contrast, paternal *H19* expression was relatively more up-regulated in the lighter kidneys of the *H19*^*+/hIC1*Δ*2*.*2*^ mice (Fig 8K). Although not statistically significant (P<0.1), a trend toward stronger *Igf2* repression was observed in the smaller with respect to the larger kidney (Fig 8I).

### Low methylation of *H19*^*hIC1*Δ*2*.*2*^ in germ cells

**Fig 9:**
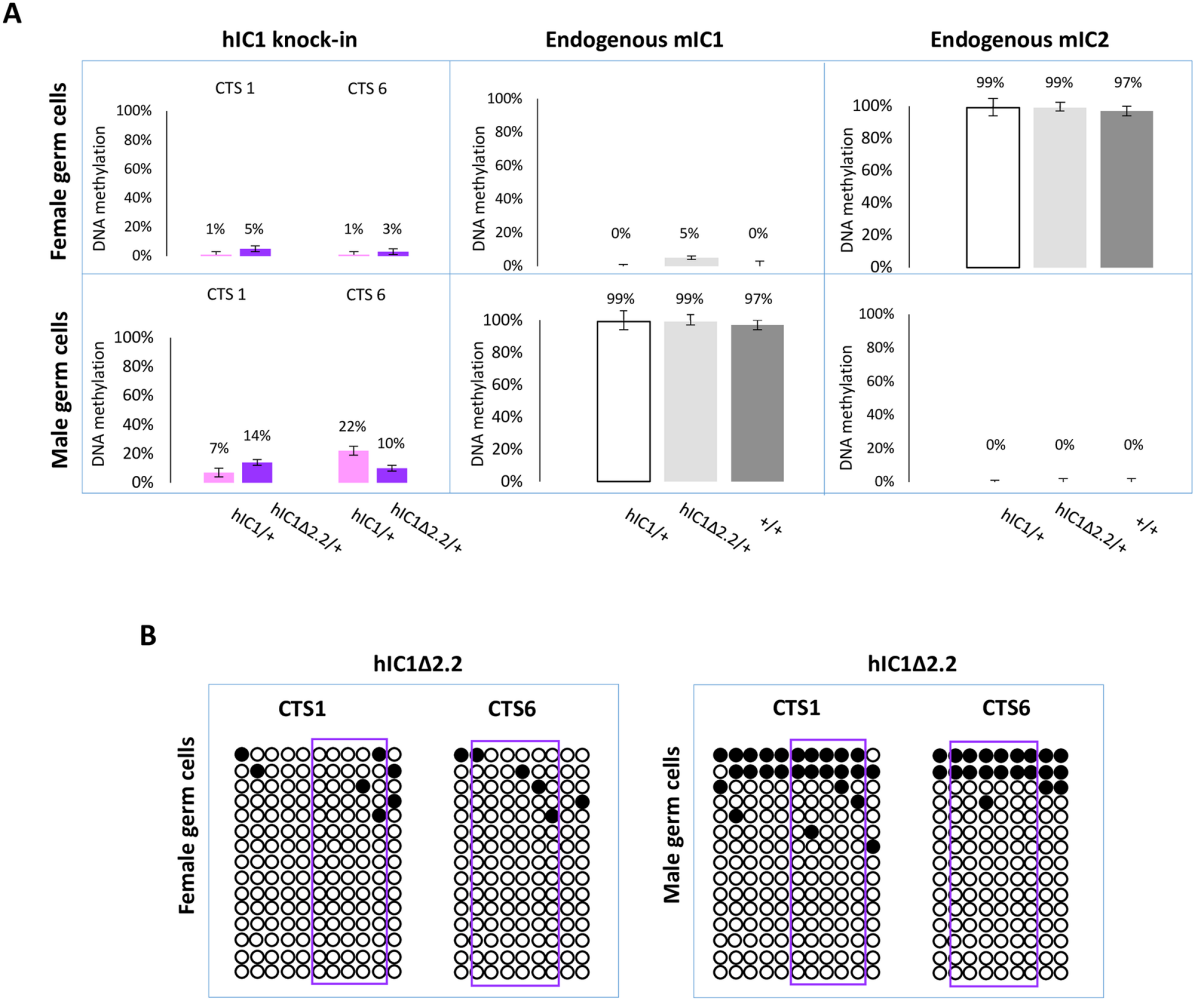
IC1 methylation in germ cells. (A) Percent methylation measured by pyrosequencing at CTS1 and CTS6 of hIC1 in oocytes and sperm of *H19*^*hIC1*Δ*2*.*2/+*^ and *H19*^*hIC1/+*^ mice (left panel). Endogenous mIC1 (middle panel) and mIC2 (right panel) were analysed as controls of the methylation assay and to rule out contamination of somatic cells. As expected, mIC1 was unmethylated in oocytes and fully methylated in sperm, and viceversa mIC2 was fully methylated in oocytes and unmethylated in sperm, in all tested mouse lines. Each histogram represents the methylation mean value of 5 (CTS1, mIC1 and mIC2) or 6 (CTS6) CpGs, tested in a pool of germ cells collected from 4–5 females and 2 males. Bars represent the mean ± SEM. (B) IC1 methylation analysed by bisulphite treatment followed by cloning and sequencing in oocytes and sperm of *H19*^*hIC1*Δ*2*.*2/+*^ mice.

Having demonstrated that *H19*^*hIC1*Δ*2*.*2*^ is methylated in somatic cells upon both maternal and paternal transmission, we asked if methylation was already present in germ cells. For this purpose, we measured DNA methylation levels in oocytes and sperm by pyrosequencing. As previously demonstrated, *H19*^*hIC1*^ remains properly hypomethylated in female gametes but methylation is inefficiently established on *H19*^*hIC1*^ in male gametes (Fig 9A and Ref. 37). Similarly, *H19*^*hIC1*Δ*2*.*2*^ methylation was close to 0% and comparable with the endogenous mIC1 in oocytes (Fig 9A). mIC2 was also analysed as control and to rule out contamination of somatic cells. The expected methylation value close to 100% was found in *H19*^*hIC1*Δ*2*.*2/+*^, *H19*^*hIC1/+*^ and *H19*^*+/+*^ oocytes.

In sperm, methylation levels were relatively low (10–14%) on *H19*^*hIC1*Δ*2*.*2*^ as well as *H19*^*hIC1*^ (7–22%), while the endogenous mIC1 was almost 100% methylated (Fig 9A). The endogenous mIC2, analysed as control, was correctly unmethylated in all three lines (*H19*^*hIC1*Δ*2*.*2/+*^, *H19*^*hIC1/+*^ and *H19*^*+/+*^). The low methylation status of *H19*^*hIC1*Δ*2*.*2*^ was confirmed by bisulphite sequencing, in both oocytes and sperm (Fig 9B).

Overall, these results demonstrate that while DNA methylation of mutant hIC1 is normally absent in oocytes, methylation is not efficiently established on the mutant KI as well as the wildtype KI in male germ cells, indicating that paternal hypomethylation of the *H19*^*hIC1*Δ*2*.*2*^ allele is seemingly acquired as early as germline development and persists into embryo development, while maternal hypermethylation of hI^C1Δ2.2^ alleles does not occur until after fertilization.

## Discussion

Gain and loss of IC1 methylation result in *H19/IGF2* imprinting defects that are characteristic of BWS and SRS, respectively. In some patients, genetic mutations have been found associated with DNA methylation abnormalities *in cis*. However, the mechanism by which the genotype affects the epigenotype and phenotype in these cases is unknown. By employing a KI mouse line, this study demonstrates that a human genetic IC1 mutation reproduces several molecular and phenotypic abnormalities of BWS and SRS. The analysis of this mouse model provides mechanistic insights into the origin of prenatal overgrowth and undergrowth associated with *H19/Igf2* imprinting defects, which are useful for understanding the aetiopathogenesis of BWS and SRS.

We have previously demonstrated that maternal transmission of hIC1 can functionally replace mIC1 in the mouse by properly regulating IC1 methylation and *H19/Igf2* imprinting [37]. We now observe that mice maternally inheriting the mutant KI allele acquire methylation on hIC1Δ2.2 after fertilization, exhibit *H19* repression and biallelic *Igf2* activation, and pre- and post-natal overgrowth. Upon paternal transmission, hIC1 lacks methylation resulting in complete *Igf2* silencing, *H19* activation, severe growth restriction particularly in placenta and embryonic lethality [37]. In contrast, paternal hIC1Δ2.2 is partially methylated and results in a more moderate imprinting defect and pre/post-natal undergrowth which is compatible with life.

Methylation is established only in a minority of male germ cells on both hIC1Δ2.2 and hIC1 and appears unstable indicating evolutionarily divergent mechanisms of imprinting establishment between human and mouse [37]. However, while hIC1 is completely unmethylated, hIC1Δ2.2 shows partial methylation on both paternal and maternal chromosomes in embryo and placenta. Thus, methylation is likely acquired *de novo* and in mosaic form on hIC1Δ2.2 in somatic cells, during development. The functional difference between hIC1Δ2.2 and hIC1 is likely due to the lack of three CTSs in the mutant allele, which shows lower affinity for CTCF in human cells [36]. The results are consistent with hIC1 having an intrinsic propensity to acquire methylation that is inhibited by CTCF binding [32]. The different behaviour of hIC1Δ2.2 and mIC1, which share a similar number of CTSs (4) and ZFP57 binding sites (6), suggests that the CTSs alone are not sufficient to maintain insulator function and that CTS spacing or other transcription factor binding sites contribute to IC1 function. It is possible that one or more of these elements are reduced or missing and this exposes the mutant hIC1 allele to the action of *de novo* DNA methyl-transferases in pre- and/or post-implantation embryos.

The mechanisms by which hIC1 methylation is altered in BWS and SRS are unknown. Our mouse model indicates that the maternal hIC1Δ2.2 methylation is acquired post-zygotically in BWS, but does not allow to distinguish if the partial IC1 methylation of SRS is due to a primary germ cell imprint establishment defect, or a post-zygotic maintenance defect, or both. However, the observation that the hIC1 methylation status can drastically change from gametes to somatic cells, suggests that maintenance mechanisms have a critical role in the origin of imprinting defects on both maternal and paternal chromosomes.

**Fig 10:**
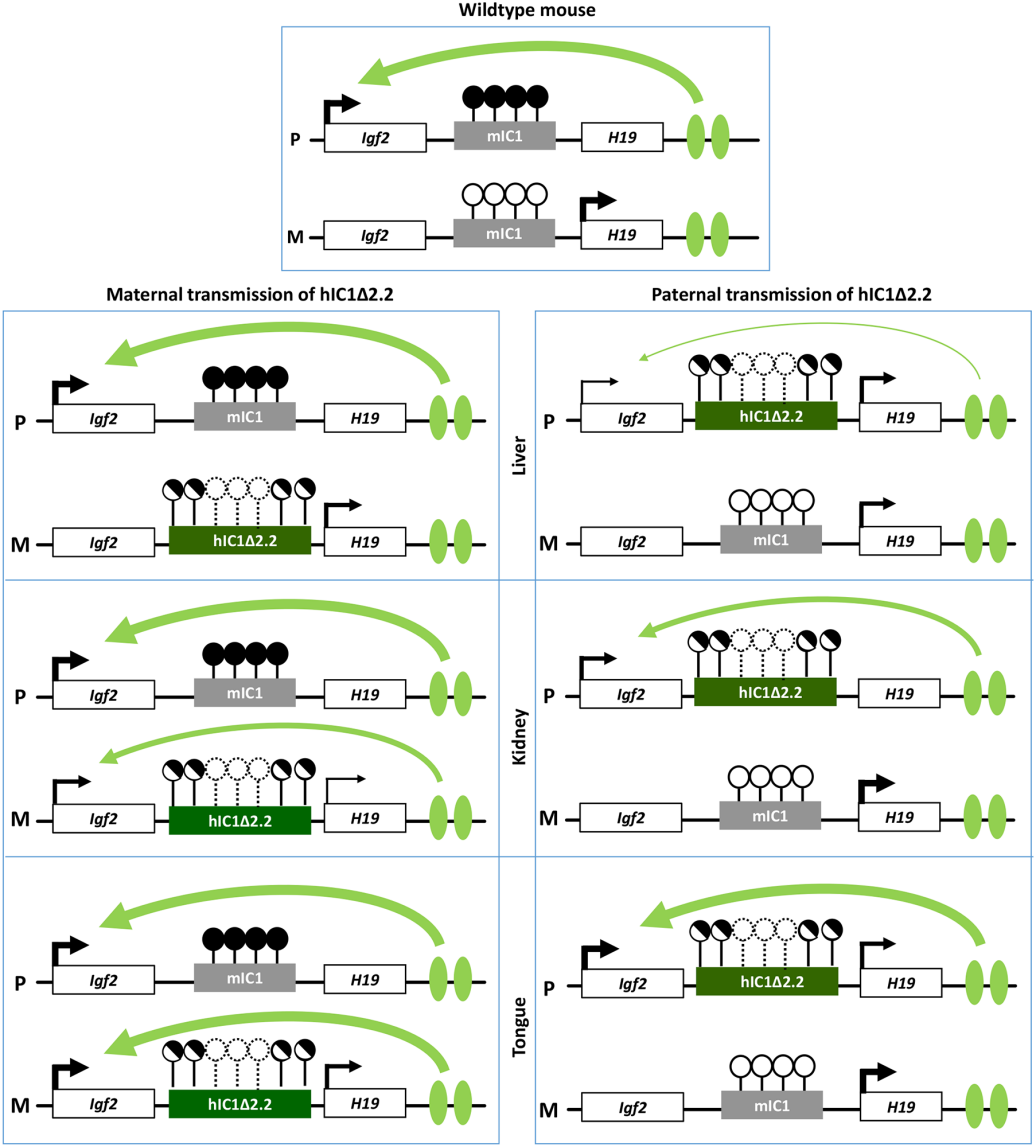
Diagram summarizing the tissue-specific imprinting defects of the *H19*^*hIC1*Δ*2*.*2*^ mice. White rectangles represent the *Igf2* and *H19* genes, grey rectangle mIC1 and green rectangles hIC1Δ2.2. Enhancers are indicated by green ovals. Transcription direction is indicated by arrows and transcription intensity by arrow thickness. Lollipops indicate CTS methylation status: filled lollipops, methylated CTSs; open lollipops: non-methylated CTSs; half-filled lollipops: partially methylated CTSs; dashed lollipops: missing CTSs in the deleted region of hIC1Δ2.2. Curved arrows indicate *Igf2* activation by downstream enhancers: arrow thickness is inversely proportional to the insulator strength inferred from allele-specific *Igf2* expression in each specific tissue. P: paternal chromosome; M: maternal chromosome.

Molecular analyses show that, although hIC1Δ2.2 is similarly methylated in neonatal liver, kidney and tongue, allele-specific *H19*/*Igf2* expression is differently altered, suggesting that hIC1 enhancer blocking function is regulated in a tissue-specific manner (Fig 10), consistent with what was previously shown for mIC1 [35]. In particular, the insulator activity of hIC1Δ2.2 appears to be robust in liver and placenta, as demonstrated by weak expression of the *Igf2* allele in *cis* with the KI. In contrast, the relatively high *Igf2* expression indicates that the insulator activity of hIC1Δ2.2 is weak in tongue. The kidney shows intermediate and mosaic insulator activity resulting in differential *Igf2* expression between left and right organs (see below). Tissue-specific differences in insulator activity may result from different post-translational modifications of CTCF [35]. Importantly, the observation that *Igf2* expression is properly regulated in specific tissue contexts in the presence of abnormal IC1 methylation paves the way to new exciting avenues for BWS and SRS therapy. Concerning *H19*, this gene is significantly down-regulated in all three tissues of *H19*^*hIC1*Δ*2*.*2/+*^ mice and up-regulated in liver and tongue of *H19*^*+/hIC1*Δ*2*.*2*^ mice, with respect to wildtype littermates, consistent with the partial methylation of hIC1Δ2.2 and the demonstrated repressor activity of methylated IC1 [32]. In liver of *H19*^*+/hIC1*Δ*2*.*2*^ neonates, a strong derepression of the imprinted allele was not accompanied by a global (mat + pat) increase of the *H19* RNA, possibly because of other physiological perturbations limiting its expression.

Overall, gene expression results are consistent with the hypothesis that the organomegaly of *H19*^*hIC1*Δ*2*.*2/+*^ mice is associated with autocrine/paracrine effects of IGF2, while defective hepatic and placental IGF2 expression underlie the growth restriction of *H19*^*+/hIC1*Δ*2*.*2*^ mice. Opposite deregulation of the growth inhibitory *H19* transcript is likely playing an additional role in the growth abnormalities of the mice with maternal and paternal transmission of the mutant KI.

Although in humans this mutation is associated only with BWS, the hIC1Δ2.2 mice display several features of BWS and SRS, on maternal and paternal transmission of the KI, respectively. Growth abnormalities originate prenatally and persist in adulthood. In particular, *H19*^*+/hIC1*Δ*2*.*2*^ mice do not catch-up growth during development, as seen in the majority of children with SRS [38]. In addition, nephromegaly and macroglossia that are observed in *H19*^*hIC1*Δ*2*.*2/+*^ mice are also distinctive clinical signs of BWS with IC1 molecular defects [39]. Finally, the tissue-specific differences of hIC1 function we observed in the mouse may explain some of the aetiopathogenetic mechanisms of BWS and SRS. The model we propose predicts that maternal IC1 GOM in BWS causes *IGF2* activation primarily in peripheral tissues, such as tongue and kidney resulting in macroglossia and organomegaly, while paternal IC1 LOM in SRS causes *IGF2* repression primarily in liver and placenta leading to deficient growth stimulation through defective endocrine secretion and placenta function.

Both *H19*^*hIC1*Δ*2*.*2/+*^ and *H19*^*+/hIC1*Δ*2*.*2*^ mice show differential growth of the kidneys indicating that asymmetric growth of bilateral organs occurs in these animals, as in many cases of BWS with IC1 defects [40]. Derepression of the maternal *Igf2* allele and repression of the paternal *Igf2* allele are incomplete in the kidney of P1 *H19*^*hIC1*Δ*2*.*2/+*^ and P1 *H19*^*+/hIC1*Δ*2*.*2*^ mice, respectively (see Figs 3 and 6), indicating mosaic expression of *H19/Igf2* in these tissues. Further analyses showed that, although IC1 methylation and *H19* RNA levels were similar, *Igf2* expression was higher in the larger than in the smaller kidney due to imprinting defects, indicating that *Igf2* is mostly responsible for the asymmetric kidney growth. These results raise the hypothesis that mosaic *IGF2* expression may also cause lateralized somatic overgrowth in humans. Our results are consistent with the findings of Ginart *et al* [41] demonstrating that the incomplete derepression of the paternal *H19* allele in mutant mice can result from an epigenetic mosaicism at a single cell level.

Overall, our findings show that a mutant human IC1 sequence can reproduce the opposite growth and molecular phenotypes of BWS and SRS in mouse, when introduced at the orthologous locus. Several mouse lines carrying mutations in the *H19/Igf2* locus have been described so far. Some of these mice, including a 1.3 kb deletion of mIC1 (Δ2,3) that results in tissue-specific loss of *Igf2* imprinting, show similarities with our *H19*^*hIC1*Δ*2*.*2*^ model [28, 34–35]. However, the methylation defects, growth abnormalities and *H19/Igf2* dysregulation of *H19*^*hIC1*Δ*2*.*2*^ more closely reproduce the phenotypic features and contribute better in understanding the molecular pathogenesis of BWS and SRS. Such humanized mouse models will be useful for more accurately unravelling pathogenetic mechanisms and for developing new therapeutic strategies in these rare congenital growth disorders.

## Materials and methods

### Targeting of the mIC1 locus and generation of the KI mice

To generate the *H19*^*hIC* Δ*2*.*2*^ mouse line, we performed gene targeting by homologous recombination in E14 embryonic stem (ES) cells [42] to target the endogenous mIC1 with a plasmid containing the *H19*^*hIC1*Δ*2*.*2*^ allele and neomycin resistance cassette (NeoR; Fig 1A). Briefly, a *PciI*–*MluI* restriction fragment of 620 bp spanning the break-point of the 2.2 kb deletion found in a BWS family [18] was extracted from the EΔ2.2 (B5/b1)pL vector [36] and subcloned in the Δ3.8kb-5’ pre-targeting vector containing the wildtype hIC1 region [28]. Sanger sequencing of the fragment was performed and no variant was found in respect to the reference human genome hg19. The subsequent steps to obtain the targeting vector were performed as previously described [37]. To compare *H19*^*hIC1*^ and *H19*^*hIC1*Δ*2*.*2*^ in the same strain background and avoid animal transfer from US to Italy, the hIC1 KI line was generated anew by performing gene targeting in E14 ES cells with the original vector used in the previous study [37].

Injection into B6 blastocysts of the *H19*^*hIC1-neo*^ and *H19*^*hIC1*Δ*2*.*2-neo*^ KI ES clones and generation of chimeras were performed by Cogentech Facility S.c.a.r.l. (Milan, Italy). Chimeras were crossed to B6 mice and germline transmission of the KI was confirmed in the agouti progeny by PCR-genotyping using primers flanking the deletion break point (hDMDB5SeqF: 5’–GGTAGTGAGGGATAGAACAC– 3’; hDMDB1RepR: 5’–GAGTGTCCTATTCCCAGATGAC– 3’) (Fig 1B). The NeoR cassette was excised by crossing heterozygous KI with pCX-NLS- Cre transgenic mice [43] on a B6 background. Excision was tested by PCR using primers flanking the NeoR cassette (NeoEXL3: 5’–ACAGAATCGGTTGTGGCTGT– 3’ H19SeqR1: 5’–CCACAGAGTCAGCATCCAC– 3’) (Fig 1C). KI mice without the NeoR cassette were crossed with B6 mice and only the progeny carrying the KI and lacking the Cre gene were selected to expand and assay the KI lines. All animal experimentation was conducted in accordance with the guidelines of the Animal Care and Use Committee of Campania University “Luigi Vanvitelli” (Naples, Italy) and was authorized by the Italian Ministry of Health.

### Extraction of nucleic acids

Liver, kidneys and tongue were collected from mice at birth and at 14 weeks of age. Placenta and whole body excluding the head were recovered from conceptuses of 15.5 days post coitum (E15.5). Genomic DNA was isolated from tissues following the standard protocol of proteinase K digestion and phenol-chloroform extraction. Total RNA was extracted using TRI Reagent (Sigma-Aldrich Italia, Milan) and following the manufacturer’s protocol. Concentrations of nucleic acids were determined with Nanodrop spectrophotometer. Sperm was isolated from adult mice and DNA was extracted as described previously [44]. Unfertilized oocytes were collected from 4–5 superovulated females of 8 weeks and resuspended in 0.03% SDS, 10 mg glycogen, 10 mg proteinase K and 1 x PBS to a final volume of 20μl. Suspension was incubated at 37°C for 90 min followed by 15 min at 95°C before sodium bisulphite treatment for DNA methylation analysis (see below).

### Gene expression analysis

About 1 μg of total RNA was treated with RNase-free DNase, and first-strand cDNA was synthesized using the QuantiTech Reverse Transcription Kit (Qiagen Italy, Milan), according to the manufacturer’s protocol. Total expression of *H19* and *Igf2* was measured by SYBR Green quantitative real-time RT-PCR (Applied Biosystems Italy, Milan). Reactions were set up in triplicate and run on ABI PRISM 7500 using the default cycling conditions. Relative expression was determined using the ΔΔC_T_ method, and the gene expression values were normalized to the expression of the *Gapdh*, *Arpp0* and *beta-actin* reference genes. Primer sequences are available on request. Allele-specific expression analysis was performed by typing for the polymorphisms present in the F1 progeny between the C57BL/6 (B6) and Balb/C mouse strains. The *Msp*I Restriction Fragment Length Polymorphism (RFLP) of *H19* (GRCm38/mm10 chr 7:142,577,609; sequenced region: chr7:142,577,530–142,577,732) and the (CA)_n_ repeat of *Igf2* (GRCm38/mm10 chr 7:142,652,936–142,652,973; sequenced region: chr7:142,652,821–142,653,091) were analysed as described by Pedone *et al*, [45]. The forward primer of *Igf2* was labelled with FAM and the PCR products were run on the ABI 3130XL fluorescent capillary system (Applied Biosystems Italy, Milan).

### DNA methylation analysis

The methylation status of cytosines in gDNA was determined by bisulphite treatment followed by pyrosequencing or cloning and sequencing. About 1 μg of genomic DNA was treated by sodium bisulphite using the Epitech Kit (Qiagen Italy, Milan), according to the manufacturer’s instructions. For the pyrosequencing, converted DNA was amplified with primers of which the reverse primer was biotinylated. The PCR products were run on the PyroMark Q24 platform, using PyromMark Gold Q96 Reagent [Qiagen Italy, Milan]. For bisulphite cloning and sequencing, converted DNA was amplified, the PCR products were cloned in Topo pCR2.1 vector (Topo-TA cloning kit, Termo Fisher Scientific Italy, Milan) and the clones were sequenced by Sanger method at Microtech Sequencing Core (Naples, Italy). Primers and PCR conditions are reported in S1 Table.

### Statistical analyses

Unless otherwise indicated, data are expressed as the mean ± standard error of the mean (SEM). The significance of the difference between two groups (KI and wildtype) was determined with a two-tailed Student’s t-test with two-sample unequal variance. The number of samples, animals, biological or technical replicates are indicated in the respective figure legends. Differences with P-values ≤ 0.05 were considered significant.

## Supporting information

S1 Fig (Related to Fig 2). Phenotypic characterization of *H19*^*hIC1/+*^ mice. Box plots of birth weights (A), growth charts (B), and box plots of organ weights (C) of *H19*^*hIC1/+*^ mice and *H19*^*+/+*^ littermates. Box plots in (A) and (C) and growth chart in (B) are depicted as in Fig 2. Values in brackets indicate the number of animals derived from two (A and C) or three (B) litters that was used for this study. P = NS: Not Significant. Bars represent the mean ± SEM.
(TIF)

S2 Fig (Related to Fig 3). Analysis of *H19* and *Igf2* expression in *H19*^*hIC1/+*^ newborn mice. (A) Allele-specific expression of *H19* and *Igf2* analysed as in Fig 3. (B) Histograms of total *H19* and *Igf2* expression in three different neonatal organs of *H19*^*hIC1/+*^ and *H19*^*+/+*^ littermates. The animals used for this study derived from two litters.
(TIF)

S3 Fig Stability of IC1 methylation of *H19*^*hIC1*Δ*2*.*2/+*^ mice after consecutive passages through the female germline. Percent methylation measured by pyrosequencing at two CTCF target sites (CTS1 and CTS6) in three different organs collected from *H19*^*hIC1*Δ*2*.*2/+*^ mice derived from three successive generations of breeding *H19*^*hIC1*Δ*2*.*2/+*^ females with +/+ males (II, III and IV generations). Black symbols: KI mice, white symbols: +/+ mice. Tested mice derive from two litters in each generation (n = 6, 4, 5). See legend to Fig 4 for more details.
(TIF)

S4 Fig Analysis of *H19* and *Igf2* expression in *H19*^*+/hIC1*Δ*2*.*2*^ and *H19*^*+/hIC1*^ mice at E15.5. (A-B) Histograms of total *H19* and *Igf2* expression in embryo body and placenta of *H19*^*+/hIC1*Δ*2*.*2*^ (A) and *H19*^*+/hIC1*^ (B) mice at E15.5 compared to relative *H19*^*+/+*^ littermates, analysed as in Fig 3. (C-D) Allele-specific expression of *H19* and *Igf2* in in embryo body and placenta of *H19*^*+/hIC1*Δ*2*.*2*^ (C) and *H19*^*+/hIC1*^ (D) mice at E15.5. Dots indicate the percent expression of the paternal allele in each individual sample. The animals used for this study derived from two litters.
(TIF)

S5 Fig IC1 methylation in *H19*^*+/hIC1*Δ*2*.*2*^ mice. (A) See legend to Fig 4A. (B) Percent methylation measured by pyrosequencing at CTS1 and CTS6 in three different organs collected from *H19*^*+/hIC1*Δ*2*.*2*^ mice at birth (left panel). The endogenous mIC1 was analysed as in Fig 3. Each histogram represents the methylation mean value of 5 (CTS1 and mIC1) or 6 (CTS6) CpGs, tested in *H19*^*+/hIC1*Δ*2*.*2*.2^ (n = 8) and *H19*^*+/+*^ (n = 5) mice derived from three litters. Bars represent the mean ± SEM. (C) IC1 methylation analysed by bisulphite treatment followed by cloning and sequencing in the tongue of a *H19*^*+/hIC1*Δ*2*.*2*^ mouse. See legend to Fig 3 for more details.
(TIF)

S6 Fig Stability of hIC1Δ2.2 methylation through the male germline and after the passage from female to male germline. (A) Percent methylation measured by pyrosequencing at two CTCF target sites (CTS1 and CTS6) in three different organs collected from *H19*^*+/hIC1*Δ*2*.*2*^ mice of three successive generations of breeding *H19*^*+/hIC1*Δ*2*.*2*^ males with +/+ females (II, III and IV generations of the pedigree). (B) Stable methylation phenotype in mice derived from breeding of *H19*^*hIC1*Δ*2*.*2/+*^ female with +/+ male (II generation) and *H19*^*hIC1*Δ*2*.*2/+*^ male with +/+ female (III generation). Tested mice derive from two litters in each generation: in (A) *H19*^*+/hIC1*Δ*2*.*2*^ (n = 5, 5, 6); in (B) *H19*^*hIC1*Δ*2*.*2/+*^ (n = 6) and *H19*^*+/hIC1*Δ*2*.*2*^ (n = 6). See legend to Fig 4 for more details.
(TIF)

S7 Fig Absence of kidneys asymmetry in *H19*^*hIC1/+*^ mice. Weight ratio of the heavier to the lighter kidney in *H19*^*hIC1/+*^ and their +/+ littermates derived from two litters at 12 weeks-old mice.
(TIF)

S1 Table Primers and PCR conditions of the pyosequencing assay. hIC1-:primers to amplify the human IC1 of the knock-in. mIC-: primers to amplify the mouse IC1 and IC2. F (Forward), R (Reverese): PCR primers. Seq: primers for sequencing; Btn: 5’ biotinylated primer.
(DOCX)
