# Supplementary figures and images for: Tissue-specific and mosaic imprinting defects underlie opposite congenital growth disorders in mice
Source: PLoS Genet. 2018 Feb 22;14(2):e1007243. doi: 10.1371/journal.pgen.1007243 (PMC5839592; doi:10.1371/journal.pgen.1007243)

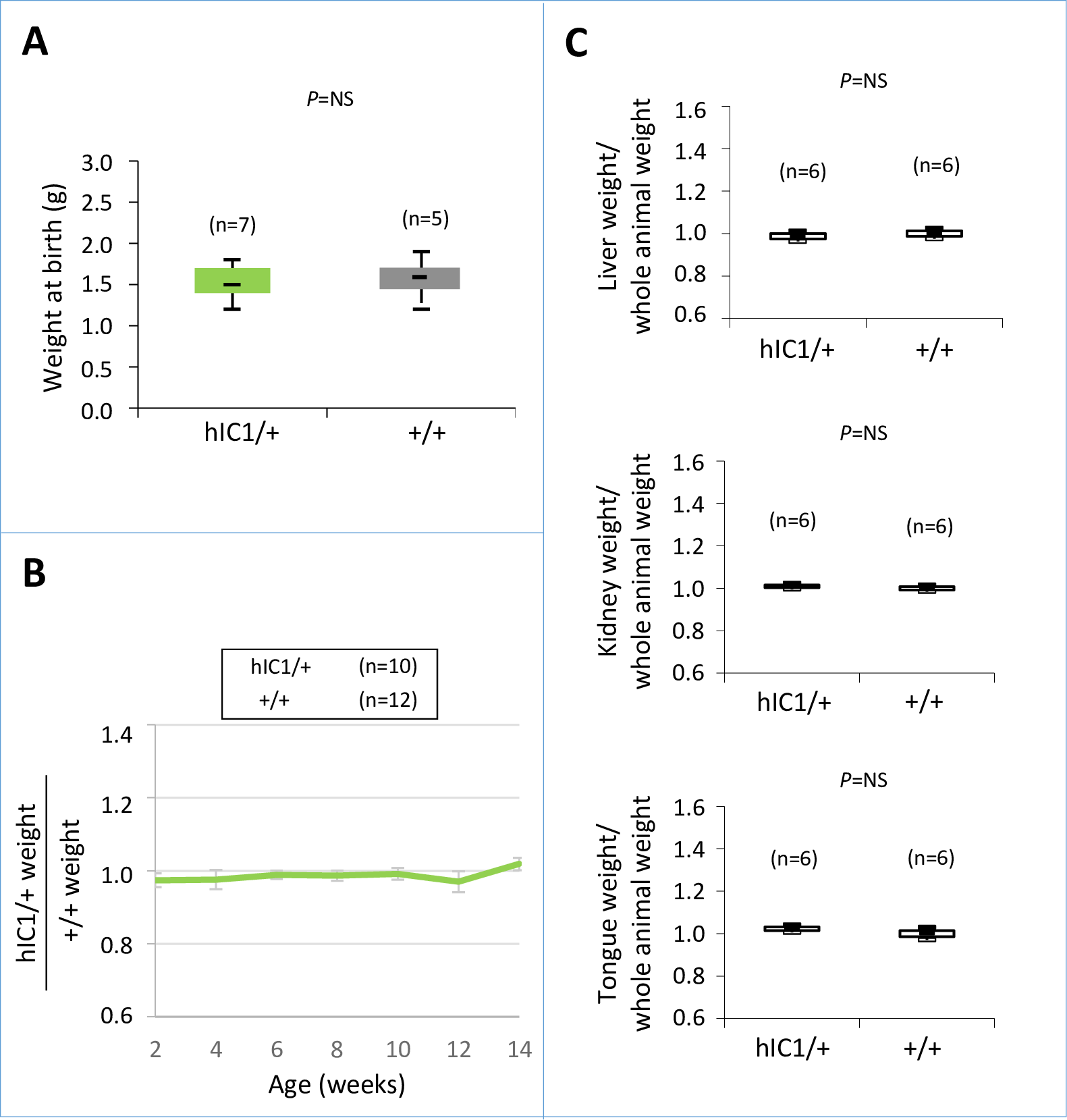

Supplement: S1 Fig — Box plots of birth weights (A), growth charts (B), and box plots of organ weights (C) of H19hIC1/+ mice and H19+/+ littermates. Box plots in (A) and (C) and growth chart in (B) are depicted as in Fig 2. Values in brackets indicate the number of animals derived from two (A and C) or three (B) litters that was used for this study. P = NS: Not Significant. Bars represent the mean ± SEM. (TIF) [file pgen.1007243.s001.tif]

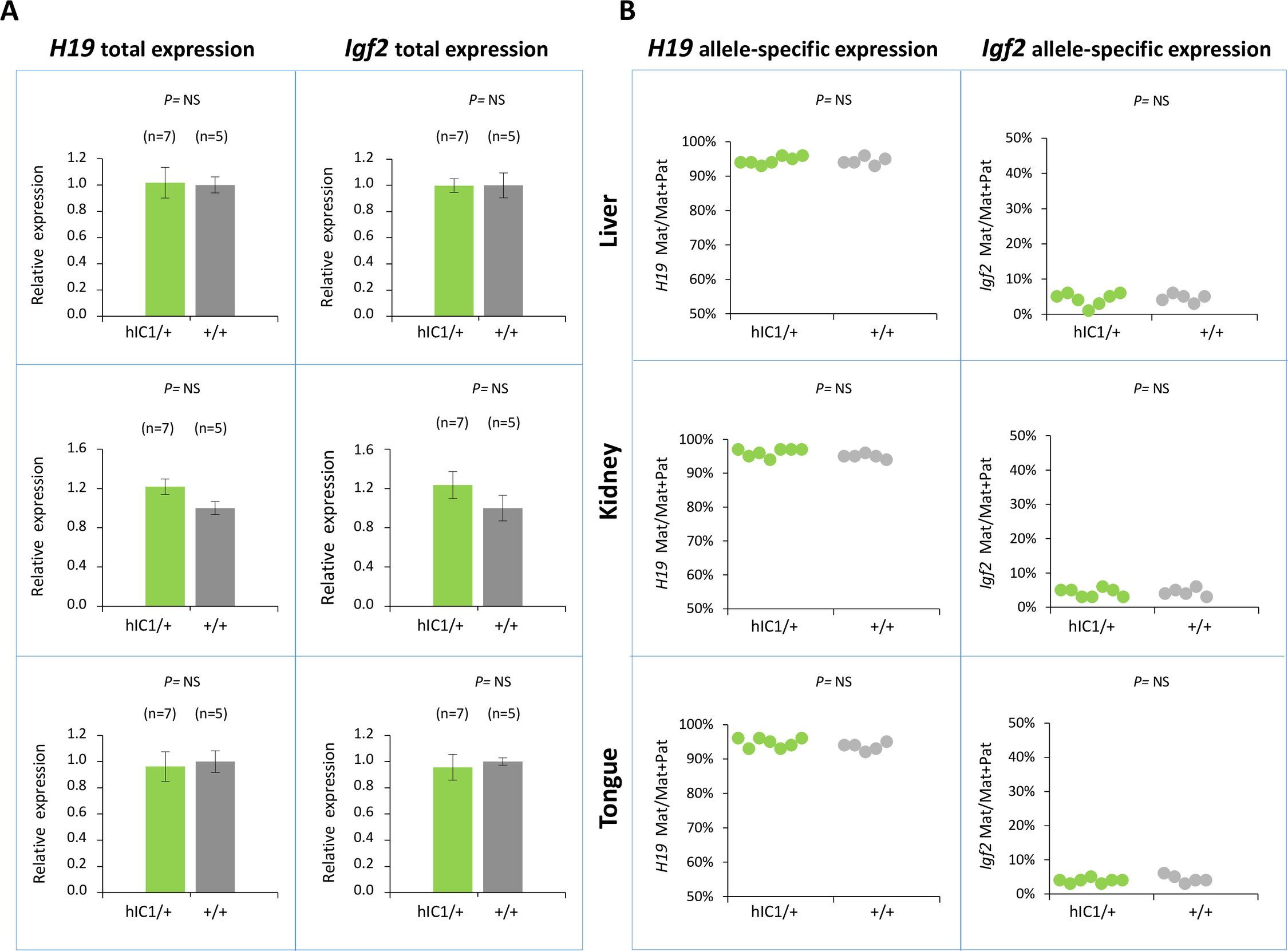

Supplement: S2 Fig — (A) Allele-specific expression of H19 and Igf2 analysed as in Fig 3. (B) Histograms of total H19 and Igf2 expression in three different neonatal organs of H19hIC1/+ and H19+/+ littermates. The animals used for this study derived from two litters. (TIF) [file pgen.1007243.s002.tif]

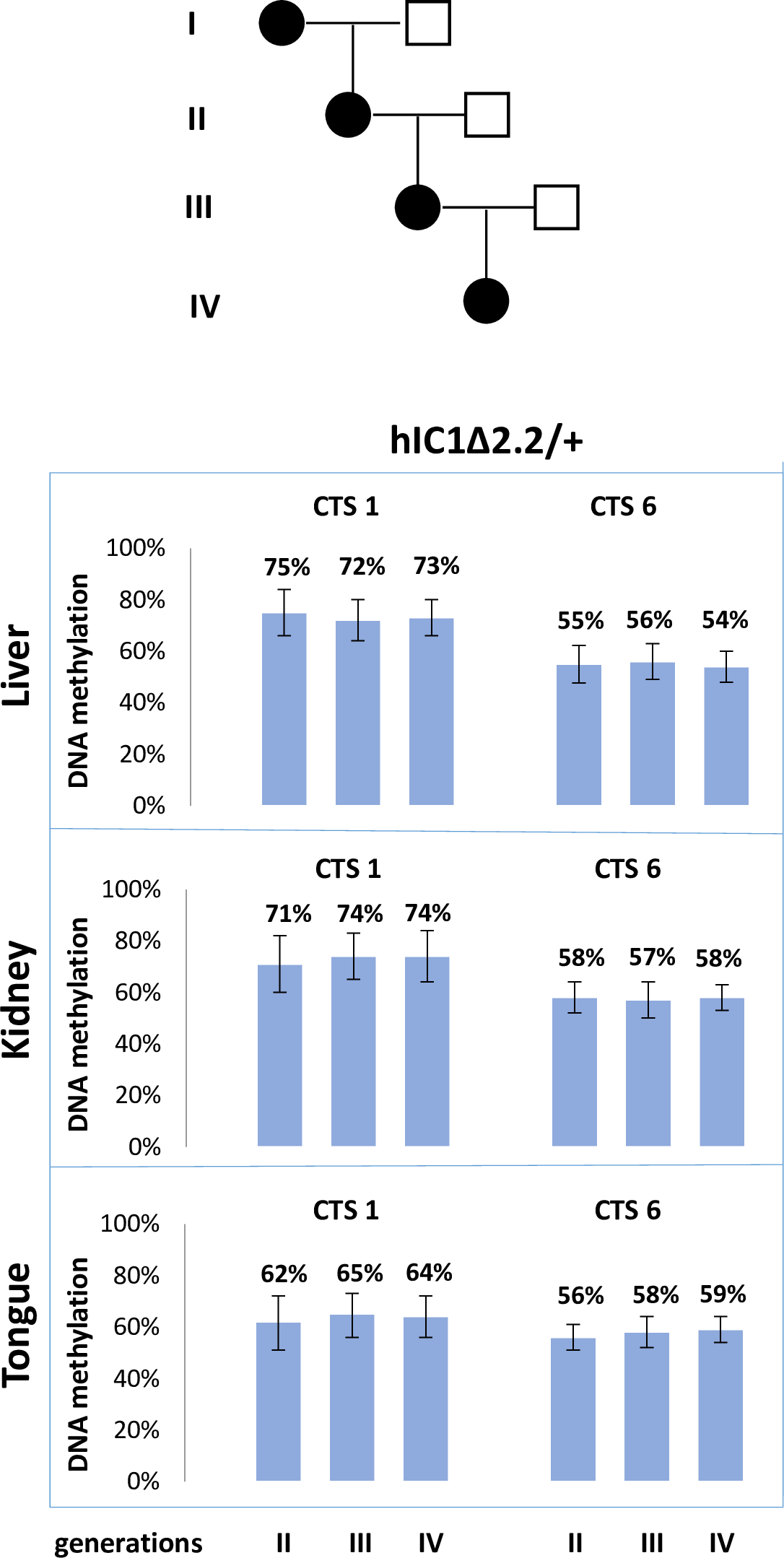

Supplement: S3 Fig — Percent methylation measured by pyrosequencing at two CTCF target sites (CTS1 and CTS6) in three different organs collected from H19hIC1Δ2.2/+ mice derived from three successive generations of breeding H19hIC1Δ2.2/+ females with +/+ males (II, III and IV generations). Black symbols: KI mice, white symbols: +/+ mice. Tested mice derive from two litters in each generation (n = 6, 4, 5). See legend to Fig 4 for more details. (TIF) [file pgen.1007243.s003.tif]

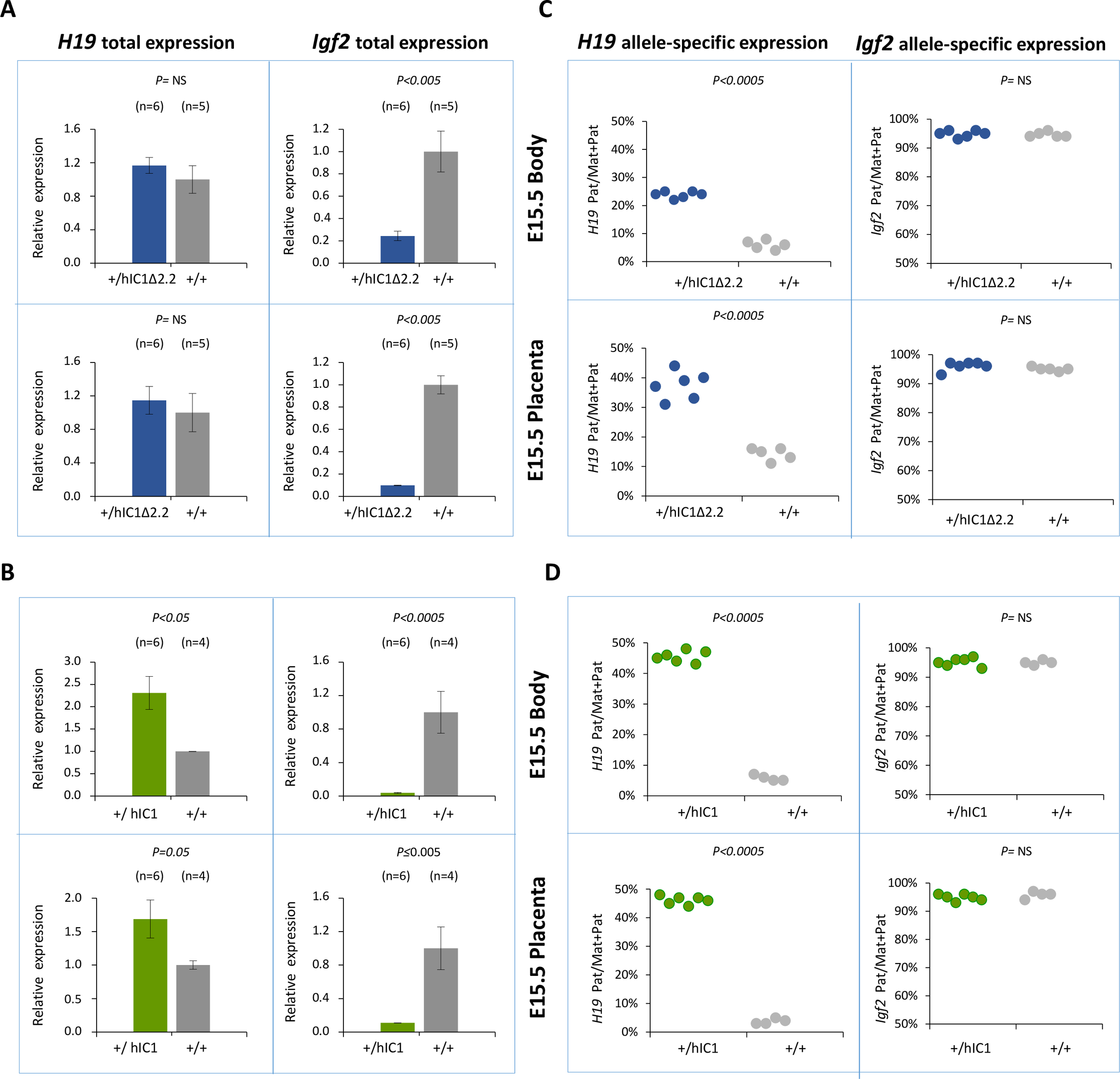

Supplement: S4 Fig — (A-B) Histograms of total H19 and Igf2 expression in embryo body and placenta of H19+/hIC1Δ2.2 (A) and H19+/hIC1 (B) mice at E15.5 compared to relative H19+/+ littermates, analysed as in Fig 3. (C-D) Allele-specific expression of H19 and Igf2 in in embryo body and placenta of H19+/hIC1Δ2.2 (C) and H19+/hIC1 (D) mice at E15.5. Dots indicate the percent expression of the paternal allele in each individual sample. The animals used for this study derived from two litters. (TIF) [file pgen.1007243.s004.tif]

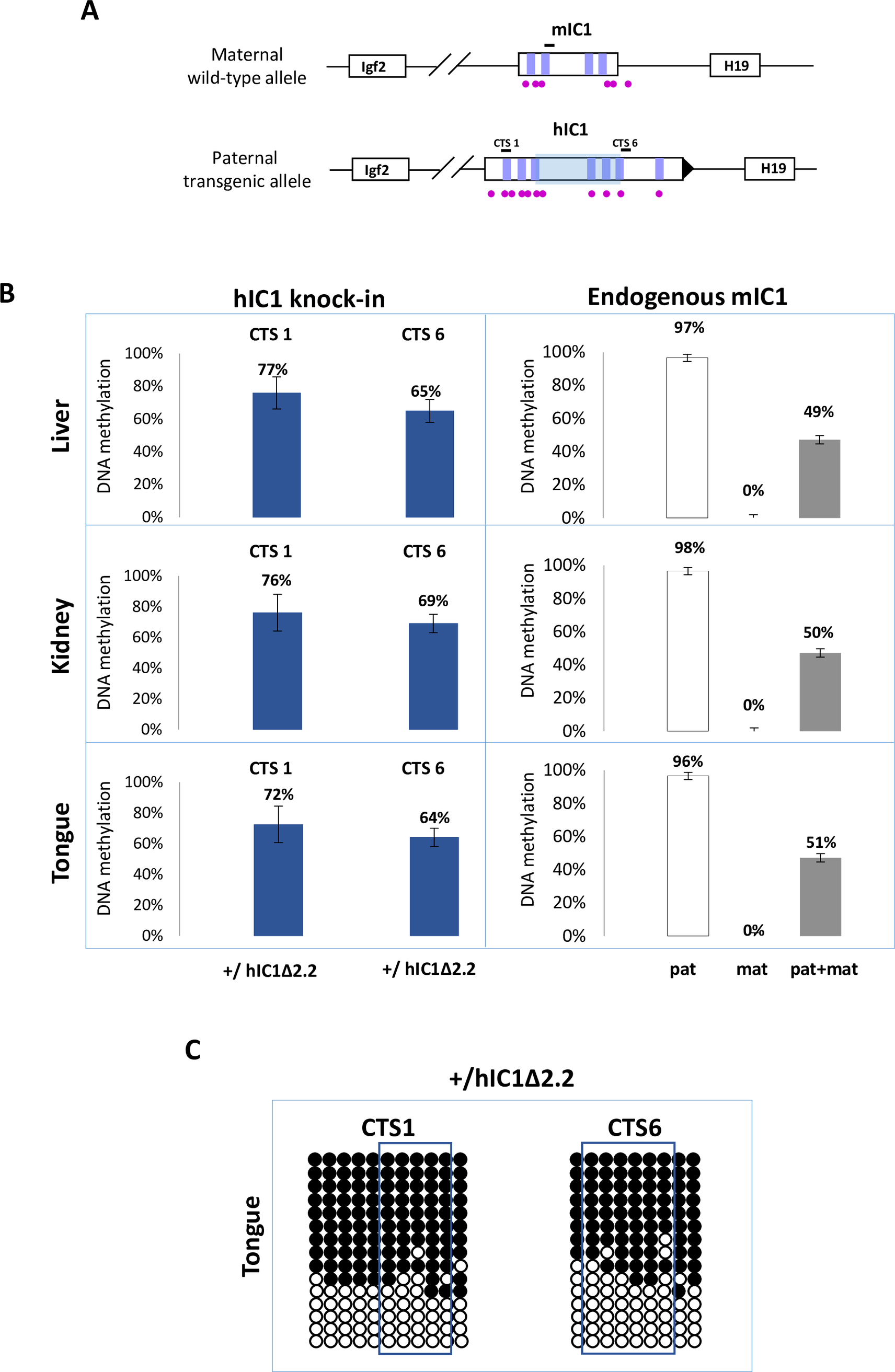

Supplement: S5 Fig — (A) See legend to Fig 4A. (B) Percent methylation measured by pyrosequencing at CTS1 and CTS6 in three different organs collected from H19+/hIC1Δ2.2 mice at birth (left panel). The endogenous mIC1 was analysed as in Fig 3. Each histogram represents the methylation mean value of 5 (CTS1 and mIC1) or 6 (CTS6) CpGs, tested in H19+/hIC1Δ2.2.2 (n = 8) and H19+/+ (n = 5) mice derived from three litters. Bars represent the mean ± SEM. (C) IC1 methylation analysed by bisulphite treatment followed by cloning and sequencing in the tongue of a H19+/hIC1Δ2.2 mouse. See legend to Fig 3 for more details. (TIF) [file pgen.1007243.s005.tif]

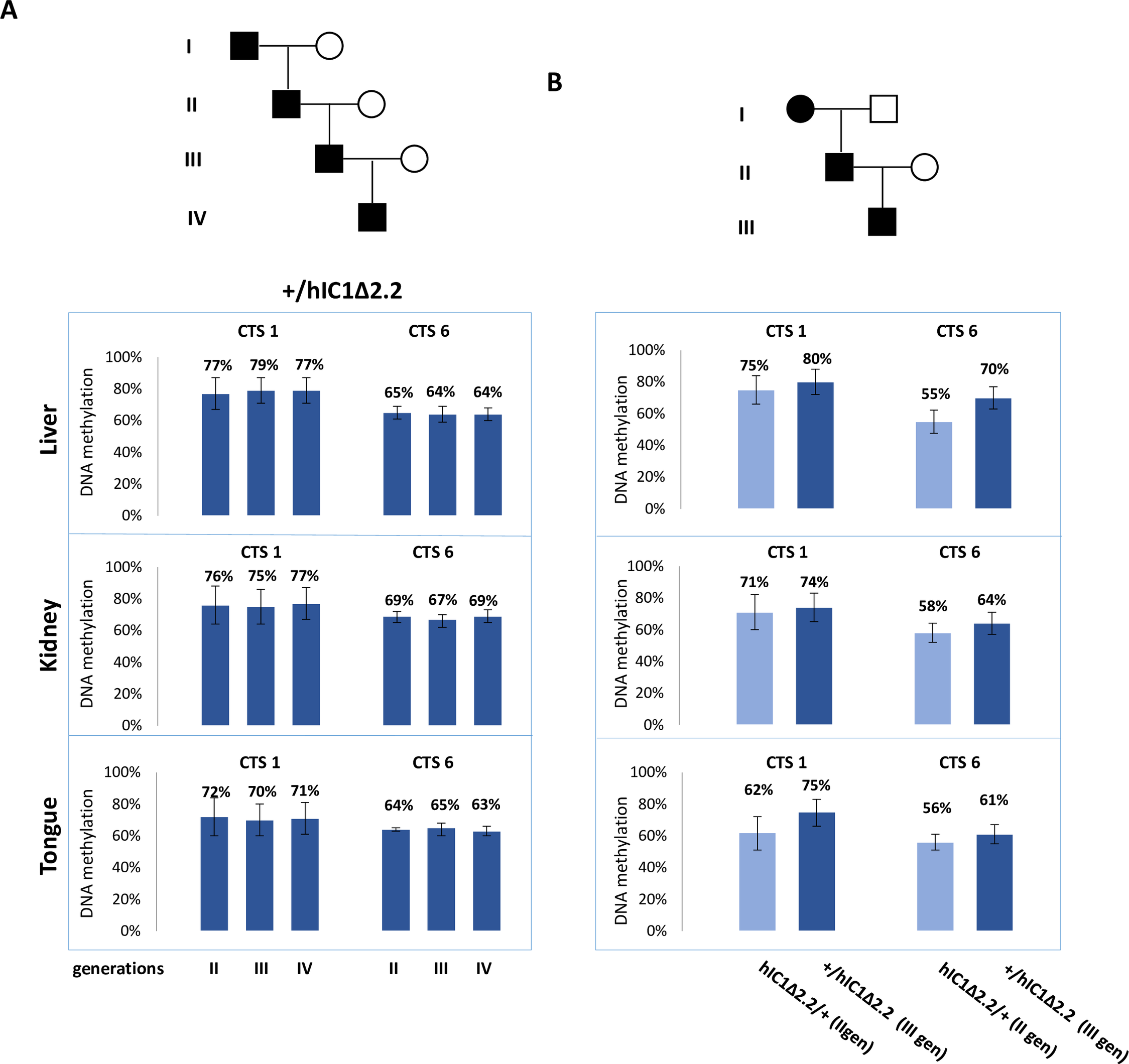

Supplement: S6 Fig — (A) Percent methylation measured by pyrosequencing at two CTCF target sites (CTS1 and CTS6) in three different organs collected from H19+/hIC1Δ2.2 mice of three successive generations of breeding H19+/hIC1Δ2.2 males with +/+ females (II, III and IV generations of the pedigree). (B) Stable methylation phenotype in mice derived from breeding of H19hIC1Δ2.2/+ female with +/+ male (II generation) and H19hIC1Δ2.2/+ male with +/+ female (III generation). Tested mice derive from two litters in each generation: in (A) H19+/hIC1Δ2.2 (n = 5, 5, 6); in (B) H19hIC1Δ2.2/+ (n = 6) and H19+/hIC1Δ2.2 (n = 6). See legend to Fig 4 for more details. (TIF) [file pgen.1007243.s006.tif]

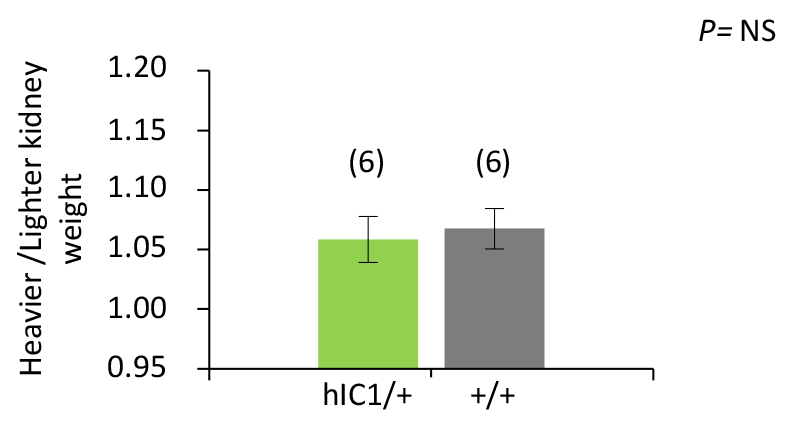

Supplement: S7 Fig — Weight ratio of the heavier to the lighter kidney in H19hIC1/+ and their +/+ littermates derived from two litters at 12 weeks-old mice. (TIF) [file pgen.1007243.s007.tif]
