# Supplementary material for: Tissue-specific and mosaic imprinting defects underlie opposite congenital growth disorders in mice
Source: PLoS Genet. 2018 Feb 22;14(2):e1007243. doi: 10.1371/journal.pgen.1007243 (PMC5839592; doi:10.1371/journal.pgen.1007243)
Supplement: S1 Table — hIC1-:primers to amplify the human IC1 of the knock-in. mIC-: primers to amplify the mouse IC1 and IC2. F (Forward), R (Reverese): PCR primers. Seq: primers for sequencing; Btn: 5’ biotinylated primer. (DOCX) [file pgen.1007243.s008.docx]

| **Region** | | **Primer Sequence** | **Annealing**  **Temp (°C)** | **MgCl_2_**  **(mM)** |
| --- | --- | --- | --- | --- |
| hIC1-CTS1 | CTS1_PyroF  CTS1_PyroR  CTS1_PyroSeq | 5’-TATTTTGTTGATTTTATTAAGGGAG-3’  5’-[Btn]ACACCTAACCTAAAAAACCTAAAAC-3’  5’-GTGTGGAATTAGAAGTGGT-3’ | 55 | 2.5 |
| hIC1-CTS6 | CTS6_PyroF  CTS6_PyroR  CTS6_PyroSeq | 5’-GGTAGTGTAGGTTTATATATTATAGTTCGAG-3’  5’-[Btn]TCCCATAAATATCCTATTCCCA-3’  5’-AGTTYGTTTTAATTGGGGTT-3’ | 57 | 2.5 |
| mIC1 | mIC1_PyroF  mIC1_PyroR^[Ref. 37]^  mIC1_PyroSeq^[Ref. 37]^ | 5’-TTTATTTTTTGTAAAGAATTTTTTGTG-3’  5’-[Btn]CTCATAAAACCCATAACTATAAAATCAT-3’  5’-TGTAAAGACCAGGGTTGC-3’ | 55 | 2.5 |
| mIC2 | mIC2_PyroF  mIC2_PyroR  mIC2_PyroSeq | 5’-AGGTTTTGGTAGGTGGTTT-3’  5’-[Btn]CCTAACTAAACCAAAATACACCATCATA-3’  5’-GTTAGGAGGAATAGTTGTTTTA-3’ | 55 | 2.0 |

**Table 1. Primers and PCR conditions of the pyosequencing assay.**

hIC1-:primers to amplify the human IC1 of the knock-in. mIC-: primers to amplify the mouse IC1 and IC2.

F (Forward) , R (Reverese): PCR primers. Seq: primers for sequencing; Btn: 5’ biotinylated primer.
